# A diet high in sugar and fat influences neurotransmitter metabolism and then affects brain function by altering the gut microbiota

**DOI:** 10.1038/s41398-021-01443-2

**Published:** 2021-05-27

**Authors:** Yinrui Guo, Xiangxiang Zhu, Miao Zeng, Longkai Qi, Xiaocui Tang, Dongdong Wang, Mei Zhang, Yizhen Xie, Hongye Li, Xin Yang, Diling Chen

**Affiliations:** 1 School of Basic Medical Science, Guangzhou University of Chinese Medicine, Guangdong, Guangzhou 510120 China; 2 State Key Laboratory of Applied Microbiology Southern China; Guangdong Provincial Key Laboratory of Microbial Culture Collection and Application; Guangdong Open Laboratory of Applied Microbiology; Institute of Microbiology, Guangdong Academy of Sciences, Guangzhou, 510070 China; 3 Academy of Life Sciences, Jinan University, Guangdong Province, Guangzhou, 510000 China; 4 Chengdu University of Traditional Chinese Medicine, Chengdu, 610075 China; 5 The Fifth Affiliated Hospital of Guangzhou Medical University, Guangzhou, 510700 China

**Keywords:** Molecular neuroscience, Neuroscience

## Abstract

Gut microbiota (GM) metabolites can modulate the physiology of the host brain through the gut–brain axis. We wished to discover connections between the GM, neurotransmitters, and brain function using direct and indirect methods. A diet with increased amounts of sugar and fat (high-sugar and high-fat (HSHF) diet) was employed to disturb the host GM. Then, we monitored the effect on pathology, neurotransmitter metabolism, transcription, and brain circularRNAs (circRNAs) profiles in mice. Administration of a HSHF diet-induced dysbacteriosis, damaged the intestinal tract, changed the neurotransmitter metabolism in the intestine and brain, and then caused changes in brain function and circRNA profiles. The GM byproduct trimethylamine-n-oxide could degrade some circRNAs. The basal level of the GM decided the conversion rate of choline to trimethylamine-n-oxide. A change in the abundance of a single bacterial strain could influence neurotransmitter secretion. These findings suggest that a new link between metabolism, brain circRNAs, and GM. Our data could enlarge the “microbiome–transcriptome” linkage library and provide more information on the gut–brain axis. Hence, our findings could provide more information on the interplay between the gut and brain to aid the identification of potential therapeutic markers and mechanistic solutions to complex problems encountered in studies of pathology, toxicology, diet, and nutrition development.

## Introduction

Gut microbiota (GM) metabolites can potentially modulate nearly all aspects of host physiology^1^, from regulating immunity^2^ and metabolism^3^ in the gut to shaping mood and behavior^4^. These metabolites can act locally in the intestine or can accumulate up to millimolar concentrations in the serum and organs^5^. Studies have shown that formation of a gut–brain neural circuit for sensory transduction of nutrients enables the gut to inform the brain of all occurrences, and make sense of what has been eaten^6^. Recent studies have revealed that the GM is important in neurodegenerative diseases, including Alzheimer’s disease (AD)^7,8^ and Parkinson’s disease (PD), and that targeting the GM or/GM metabolites could be used to treat neurodegenerative diseases ^9,10^.

An imbalanced diet that includes a high intake of sugar and fat and insufficient dietary fiber over a long time can cause enteric dysbacteriosis. The latter increases the permeability of the intestinal mucosa, and results in abnormalities in intestinal immunity and glucolipid metabolism. At this time, the dominant bacteria can change readily. For example, Fujisaka and colleagues showed that the relative abundance of *Bifidobacterium* species and *Bacillus* species decreased in mice fed a high-fat diet, whereas the abundance of Gram-negative bacteria increased^11^. Recent studies have shown that obesity is not necessary for dysfunction of the intestinal barrier. That is, hyperglycemia is more likely to drive intestinal-barrier dysfunction and the risk of enteric infection. Hyperglycemia increases the permeability of the intestinal barrier, which provides microbes with more chances to enter the body and cause proliferation of pathogenic bacteria and focal shifts^12^. Studies have demonstrated that some bacteria can produce bioactive neurotransmitters^13,14^, and these neurotransmitters are thought to regulate the nervous system activity and behavior of the host^15,16^. Recently, O Donnell and coworkers revealed that the neuromodulator tyramine produced by commensal bacteria of *Providencia* species (which colonize the gut) bypassed the requirement for host tyramine biosynthesis and manipulated a host sensory decision in *Caenorhabditis elegans*^17^. However, how these bacteria release signals to activate the brain is not known.

Using animal models, several pathways of communication have been identified along the “gut–brain–axis”, including those driven by the immune system, vagus nerve, or by modulation of neuroactive compounds by the microbiota^18,19^. In recent years, microbiota have been shown to produce and/or consume a wide range of mammalian neurotransmitters, including dopamine, norepinephrine, serotonin, or gamma-aminobutyric acid^13,20^. Accumulating evidence in animals suggests that manipulation of these neurotransmitters by bacteria may have an impact in host physiology. Preliminary clinical studies have revealed that microbiota-based interventions can also alter neurotransmitter levels^13^. Nonetheless, substantially more work is required to determine if microbiota-mediated manipulation of human neurotransmission has physiological implications and, if so, how it may be exploited therapeutically.

We chose a diet with increased amounts of sugar and fat (i.e., a high-sugar and high-fat (HSHF) diet) to disturb the GM, then monitored the effect on pathology, neurotransmitters, metabolism, and transcription of circularRNAs (circRNAs) in mice. We aimed to identify some associations between the functions of the GM, neurotransmitters, and the brain. We also aimed to enlarge the “microbiome–transcriptome” linkage library. This approach would provide more information on the interplay between the gut and brain to aid identification of potential therapeutic markers and mechanistic solutions to complex problems encountered in studies on pathology, toxicology, diets and nutrition development.

## Materials and methods

### Animals

#### Ethical approval of the study protocol

All experimental protocols were approved by the Center of Laboratory Animals of the Guangdong Institute of Microbiology (Guangzhou, China). All efforts were made to minimize the number of animals used.

#### Preparation and treatments of mice suffering from dysbacteriosis

Adult male KM mice (18–22 g, 6 weeks) were obtained from the Center of Laboratory Animals of Guangdong Province (certificate number: SCXK [Yue] 2008-0020, SYXK [Yue] 2008-0085). They were pair-housed in plastic cages in a temperature-controlled (25 ± 2 °C) colony room at a 12-h light–dark cycle. Food and water were available ad libitum.

Mice were allocated randomly into two groups of 12: control and model. The mice in the control group were fed a standard diet. The mice in the model groups were fed a HSHF diet. Water was available freely. These treatments lasted for 3 months.

The components of the HSHF diet were 20% sucrose, 15% fat, 1.2% cholesterol, 0.2% of bile acid sodium, 10% casein, 0.6% calcium hydrogen phosphate, 0.4% stone powder, 0.4% premix, and 52.2% basic feed. Heat ratio: protein 17%, fat 17%, carbohydrate 46%.

#### Preparation and treatment of SAMP8 mice and newborn KM mice

Male SAMP8 mice (5 months; mean bodyweight, 20 ± 5 g) were purchased from Beijing HFK Bioscience (SCXK [Jing] 2014-0004). Adult KM mice (18–22 g, 16 females and 8 males, 8 weeks) were obtained from the Center of Laboratory Animal of Guangdong Province (SCXK [Yue] 2008-0020, SYXK [Yue] 2008-0085). All mice were pair-housed in plastic cages in a temperature-controlled (25 ± 2 °C) colony room with a 12-h light–dark cycle. Mice had free access to food and water. All animals were allowed to acclimatize to their surroundings for ≥1 week before initiation of experimentation.

#### Effects of TMA on Sprague–Dawley rats

Twenty male Sprague–Dawley rats (180–220 g) obtained from the Center of Laboratory Animal of Guangdong Province (SCXK [Yue] 2008-0020, SYXK [Yue] 2008-0085). They were pair-housed in plastic cages in a temperature-controlled (25 ± 2 °C) colony room at a 12-h light–dark cycle. Food and water were available ad libitum.

Male Sprague–Dawley rats were divided into two groups of 10: normal group, trimethylamine (TMA)-induced group (2 mL/kg/d of 2.5% TMA purchased from Shanghai Aladdin Biochemical Technology, Shanghai China).

#### Influences of Candida albicans and Klebsiella pneumoniae on the cholinergic system in mice

The abundance of *C. albicans* (a symbiotic opportunistic pathogen in humans) and *K. pneumoniae* was found to be high in AD patients in our other study (data not shown). We chose these pathogens to ascertain the potential routes of communication/interaction between the host and its resident bacteria on neurotransmitter metabolism and brain function. *C. albicans* and *K. pneumoniae* were administered (i.g.) to normal C57 mice by monotherapy or in combination.

Adult male C57 mice (18–22 g, 6 weeks) obtained from Center of Laboratory Animal of Guangdong Province, SCXK [Yue] 2008-0020, SYXK [Yue] 2008-0085 were pair-housed in plastic cages in a temperature-controlled (25 ± 2 °C) colony room at a 12-h light–dark cycle. Twenty C57 mice were divided into four groups: normal, *C. albicans*-treated, *K. pneumoniae-*treated, and *C. albicans* + *K. pneumoniae*-treated (*Ca* + *Kp*).

### Measurement of physiological and biochemical indices

The appearance, behavior, and fur color of animals were documented every day. Bodyweight was measured every 3 days during the period of drug administration. Blood samples were drawn by removing the eye under anesthesia (isoflurane). Serum was acquired by centrifugation and stored at −80 °C until measurement. Levels of triglycerides (TG), total cholesterol, (T-CHO) and high-density lipoprotein-cholesterol (HDL-C) were measured with commercially available kits from Nanjing Jiancheng Bioengineering Institute (Jiangsu, China). Serum and brain-tissue levels of trimethylamine-n-oxide (TMAO), and neurotransmitter levels were quantified using liquid chromatography-mass spectrometry (LC-MS).

### Histopathology and immunostaining

Brain, liver, renal, spinal marrow, spleen, and adipose tissues were removed and fixed in 4% paraformaldehyde at pH 7.4 for further pathologic observation. These tissue samples were made into paraffin sections after drawing materials, fixation, washing, dehydration, transparency, dipping in wax, and embedding. Obesity-related parameters or other related pathologic changes were measured.

The brains of animals were dissected. Four brains from each group were fixed in 4% paraformaldehyde solution and prepared as paraffin sections. Sections were stained with hematoxylin and eosin (H&E). silver, or underwent Nissl staining and TUNEL staining. Immunostaining using paraffin-embedded sections (thickness = 3 μm) and a two-step method involving a peroxidase-conjugated polymer kit (Envision^®^; DAKO, Carpinteria, CA, USA) was also done. Slides were observed under light microscopy.

### Analyses of microbiome 16S rDNA

Fresh samples of intestinal content were collected 12 h before the fasting of rats and stored at −80 °C. Microbial DNA isolated from these samples, with a total mass ranging from 1.2 ng to 20.0 ng, was stored at −20 °C. Microbial 16S rRNA genes were amplified using a forward primer (5′-ACTCCTACGGGAGGCAGCA-3′) and a reverse primer (5′-GGACTACHVGGGTWTCTAAT-3′). Polymerase chain reaction (PCR) amplification was done and the products were purified, quantified, and homogenized to form a sequencing library. The established library was firstly inspected by the library, and the qualified library was sequenced by Illumina HiSeq 2500.

### Metabolomics analysis

Aliquots of each standard solution were mixed to generate a stock standard mixture of 4 μg/mL in 50% acetonitrile. This 4 μg/mL standard mixture (100 μL) was mixed with 50 μL of sodium carbonate (100 mM) and 50 μL of 2% benzoyl chloride (BZ) or 2% benzoyl chloride-(phenyl-^13^C_6_) (^13^C_6_BZ). The reaction mixture was vortex-mixed and diluted to 2400 ng/mL for BZ derivatives and 50 ng/mL with 50% acetonitrile for ^13^C_6_BZ derivatives as the internal standard (IS) stock solution. BZ derivatives were serially diluted to 240, 120, 60, 30, 12, 3, 1.2, and 0.02 ng/mL. To prepare the standard curve, the standard BZ derivative solutions stated above were mixed isometrically with 50 ng/mL of IS stock solution to generate calibration levels covering a range of 0.01–1200 ng/mL for all analytes.

#### Samples of brain tissue

Samples of brain tissue (20 ± 1 mg) were homogenized in a 4-volume (vol/wt) precooled aqueous solution of ascorbic acid (20 mM) with TissueLyser™ JX-24 (Jingxin, Shanghai, China) beads at 30 Hz for 3 min. The homogenates were supplemented with 15-volume prechilled acetonitrile (−40 °C) and vortex-mixed for 1 min before centrifugation at 4 °C and 14,000 × g for 15 min. Derivatization was started by addition of 40 μL of 2% BZ to a mixture of 40 μL of supernatant solution and 20 μL of sodium carbonate (100 mM). Derivatized samples were mixed with IS stock solution to analyze high concentrations of neurotransmitters by ultra-high pressure-tandem mass spectrometry (UPLC-MS/MS) with an injection volume of 1 μL. The derivatized sample was diluted 10-fold, and the dilution was mixed isometricallywith ISs to analyze low concentrations of neurotransmitters by UPLC-MS/MS with an injection volume of 5 μL.

#### Samples of intestinal content

Frozen samples of intestinal content (20 ± 1 mg) were homogenized in 5-volume (vol/wt) of a precooled aqueous solution of ascorbic acid (20 mM). After sonication for 3 min, the homogenates were supplemented with 15-volume prechilled acetonitrile (−40 °C) and vortex-mixed for 1 min before centrifugation at 4 °C and 14,000 × g for 15 min. Derivatization was started by addition of 40 μL of 2% BZ to a mixture of 40 μL of supernatant and 20 μL of sodium carbonate (100 mM). Derivatized samples were mixed with IS stock solution to analyze low concentrations of neurotransmitters by UPLC-MS/MS with an injection volume of 1 μL. The derivatized sample was diluted 10-fold, and the dilution was mixed isometrically with ISs to analyze high concentrations of neurotransmitters with UPLC-MS/MS with an injection volume of 10 μL.

#### UPLC-MS/MS

UPLC-MS/MS was done on a Acquity UPLC system (Waters, Milford, MA, USA) coupled to a Triple Quad™ 5500 tandem mass spectrometer (AB Sciex, Framingham, MA, USA). Each sample or standard mixture was injected onto a UPLC BEH C18 column (100 mm × 2.1 mm, 1.7 μm; Waters) at a flow rate of 0.4 mL/min. The mobile phase consisted of 2 mM ammonium acetate with 0.1% formic acid in water (A) and acetonitrile (B). Chromatographic separation was conducted by a gradient elution program: 0 min, 1% B; 0.5 min, 1% B; 1 min, 45% B; 4 min, 65% B; 4.2 min, 100% B; 5.2 min, 100% B; 5.3 min, 1% B; 7 min, 1% B. The column temperature was 40 °C.

The analytes eluted from the column were ionized in an electrospray ionization source in positive mode. The conditions were: source temperature, 600 °C; curtain gas, 30 psi; ion source gas 1, 50 psi; ion source gas 2, 50 psi; collision gas, 8 psi; ion spray voltage, 5500 V; entrance potential, 10 V; collision cell exit potential, 14 V. Scheduled multiple reaction monitoring (sMRM) was used to acquire data in optimized MRM transition (precursor > product), declustering potential, and collision energy (Table 1). The total scan time per cycle was 0.25 s. The samples and standard-curve samples were analyzed simultaneously. AB Sciex Analyst v1.5.2 (https://sciex.com/products/software/analyst-software) was used to control instruments and acquire data using the default parameters and assist manual inspection to ensure the qualitative and quantitative accuracies of each compound. The peak areas of target compounds were integrated and outputted for quantitative calculation.

**Table 1:** Different expression of circRNAs in the brain of high sugar & fat diet induced dysbacteriosis mice (control vs model group, |Foldchange| >1.50, *p* < 0.05).

circRNA_ID	Gene	mmu_circbase_id	Foldchange	*P* Value
chr12_38147502_38190134_+	Dgkb	NA	−404.091	3.28E–06
chr10_90914692_90948672_+	Anks1b	mmu_circ_0002557	−401.022	4.29E–06
chr17_44638512_44639826_−	Runx2	mmu_circ_0006773	−356.377	5.55E–05
chr14_14745967_14757431_+	Slc4a7	NA	−244.951	0.00029
chr13_83625264_83635469_+	Mef2c	NA	−243.943	0.000387
chr14_21833800_21838043_+	Vdac2	NA	−227.906	0.000523
chr8_70251613_70252101_+	Sugp2	mmu_circ_0014931	−221.4	0.000599
chr1_155187777_155197435_+	Stx6	mmu_circ_0008281	−220.455	0.001103
chr4_106420433_106423562_+	Usp24	NA	−213.924	0.000763
chr6_110914326_110915171_+	Grm7	mmu_circ_0012931	−201.04	0.001351
chr1_156869440_156888030_−	Ralgps2	mmu_circ_0008300	−185.795	0.001516
chr17_84710290_84716715_−	Lrpprc	NA	−181.382	0.002065
chr10_5172887_5175916_−	Syne1	NA	−180.507	0.001669
chr5_23565113_23629477_–	Srpk2	NA	−179.396	0.001811
chr8_85875633_85957654_+	Phkb	mmu_circ_0015077	−174.144	0.001996
chr4_88196758_88197016_+	Focad	mmu_circ_0011840	−172.585	0.004101
chr19_5797448_5798075_−	Malat1	NA	−171.865	0.002093
chr7_63615288_63685551_+	Otud7a	NA	−168.418	0.00227
chr4_86249762_86252840_+	Adamtsl1	mmu_circ_0011813	−168.018	0.002479
chr9_10419035_10658287_−	Cntn5	NA	−167.399	0.002821
chr11_36273230_36300430_−	Tenm2	mmu_circ_0003007	−166.71	0.003624
chr9_60649479_60680171_−	Lrrc49	NA	−164.043	0.003149
chr14_32250016_32262831_+	Parg	mmu_circ_0000520	−160.076	0.002826
chr7_126169128_126174248_−	Xpo6	mmu_circ_0013871	−159.96	0.003022
chr13_97929410_97936861_−	Arhgef28	mmu_circ_0004930	−157.107	0.003519
chr5_106618070_106666845_−	Zfp644	mmu_circ_0001380	−152.411	0.003532
chr5_137325322_137325674_−	Slc12a9	NA	−149.073	0.005074
chr8_94047271_94055344_+	Ogfod1	NA	−145.577	0.006017
chr9_21934117_21935628_+	Ccdc159	NA	−145.057	0.005719
chr6_101169133_101172447_−	Pdzrn3	mmu_circ_0012894	−144.036	0.004294
chr13_8842729_8861265_−	Wdr37	NA	−141.159	0.004877
chr10_58467114_58470762_+	Ranbp2	NA	−140.656	0.004984
chr12_3865984_3873428_+	Dnmt3a	mmu_circ_0003954	−139.642	0.004835
chr6_127729409_127732820_−	Prmt8	NA	−137.936	0.004863
chr8_106981125_106981339_+	Sntb2	mmu_circ_0001722	−136.429	0.00534
chr12_111097454_111100038_+	Rcor1	mmu_circ_0003755	−135.001	0.005934
chr8_99400725_99401177_−	Cdh8	NA	−133.903	0.005448
chr18_86461041_86473843_+	Neto1	NA	−133.846	0.005985
chr13_76822972_76925525_+	Mctp1	NA	−133.839	0.006304
chr9_16021315_16031420_−	Fat3	mmu_circ_0015421	−131.089	0.006281
chr7_97031337_97040036_+	Nars2	NA	−130.142	0.005893
chr15_79542486_79543856_−	Ddx17	NA	−129.349	0.005771
chr10_119986331_120010286_+	Grip1	NA	−128.055	0.005706
chr5_141962664_141975714_+	Sdk1	mmu_circ_0012262	−127.064	0.007112
chr11_4803677_4816165_−	Nf2	NA	−124.657	0.006556
chr12_77277360_77365359_+	Fut8	mmu_circ_0004207	−123.76	0.007571
chr13_30817997_30826094_−	Exoc2	NA	−122.612	0.007436
chr3_55477804_55489896_+	Dclk1	mmu_circ_0010690	−122.425	0.006334
chr9_114705053_114709314_+	Dync1li1	mmu_circ_0015352	−119.173	0.007756
chr5_140430051_140433134_+	Eif3b	NA	−117.743	0.007017
chr9_86634645_86679952_−	Me1	NA	−115.342	0.007181
chr16_13790620_13796077_+	Rrn3	NA	−115.165	0.007354
chr4_109037974_109039914_+	Nrd1	NA	−113.931	0.007381
chr14_77365025_77441899_+	Enox1	NA	−110.46	0.008948
chr10_45648590_45649987_+	Hace1	NA	−108.435	0.008376
chr1_5095614_5135937_+	Atp6v1h	mmu_circ_0008758	−107.867	0.008232
chr7_97705588_97716539_+	Clns1a	mmu_circ_0013679	−104.857	0.008756
chr6_36894775_36916461_−	Dgki	NA	−104.678	0.00873
chr9_22643744_22659145_+	Bbs9	mmu_circ_0015468	−104.402	0.008172
chr4_128296394_128338697_+	Csmd2	mmu_circ_0011199	−103.867	0.008444
chr8_123968000_123969922_−	Abcb10	NA	−103.404	0.009222
chr2_69944299_69948510_+	Ubr3	NA	−103.071	0.009949
chr17_11635309_11673052_+	Park2	NA	−102.934	0.009596
chr4_36718866_36724085_−	Lingo2	mmu_circ_0011588	−102.814	0.009541
chr15_38498668_38514609_−	Azin1	NA	−102.811	0.008268
chr4_32826929_32828808_−	Ankrd6	NA	−101.776	0.008501
chrX_140384002_140391454_+	Frmpd3	NA	−101.259	0.008774
chr1_135394135_135400203_−	Ipo9	mmu_circ_0008200	−101.042	0.008981
chr4_132656692_132673032_+	Eya3	mmu_circ_0001277	−100.539	0.008743
chr18_43562530_43567210_−	Jakmip2	NA	−100.345	0.012209
chr19_27800116_27806908_−	Rfx3	NA	−98.2611	0.01054
chr3_86816357_86831834_−	Dclk2	NA	−95.8757	0.009228
chr12_87075077_87076505_+	Tmem63c	NA	−95.0844	0.010211
chr15_12457797_12458299_−	Pdzd2	mmu_circ_0005537	−94.292	0.009719
chr9_77164764_77181396_−	Mlip	mmu_circ_0015896	−94.1348	0.010842
chr5_124632878_124639283_+	Atp6v0a2	NA	−92.7345	0.010875
chr2_158473122_158477763_+	Ralgapb	mmu_circ_0009519	−91.3784	0.010868
chr11_4799860_4820493_−	Nf2	mmu_circ_0003054	−90.1746	0.009803
chr18_12871077_12906949_−	Osbpl1a	NA	−89.874	0.011312
chr16_56151273_56155375_+	Senp7	NA	−89.8398	0.010437
chr9_50789496_50792407_+	Alg9	NA	−88.074	0.011172
chr2_158047918_158058827_+	Rprd1b	NA	−86.9627	0.01007
chr16_38505411_38518532_−	Timmdc1	NA	−86.7884	0.010072
chr6_37048119_37058028_−	Dgki	mmu_circ_0013333	−86.0778	0.010146
chr9_59394877_59412531_−	Arih1	NA	−85.9027	0.00997
chr1_184925815_184945000_−	Mark1	NA	−85.741	0.010481
chr4_58861524_58875573_−	AI314180	mmu_circ_0011714	−84.1099	0.011083
chr9_24473773_24485101_−	Dpy19l1	NA	−83.2237	0.010312
chr1_143667624_143677855_−	Cdc73	mmu_circ_0008242	−82.7125	0.010813
chr8_68460923_68461851_−	Csgalnact1	mmu_circ_0014912	−82.7124	0.010799
chr6_148411037_148425984_−	Tmtc1	mmu_circ_0013190	−81.6378	0.010321
chr4_155626773_155628823_+	Cdk11b	mmu_circ_0011499	−80.5751	0.011413
chr17_87678207_87680078_+	Msh2	NA	−80.3893	0.010289
chr2_6721416_6747946_−	Celf2	NA	−80.0149	0.012001
chr9_96287600_96310688_+	Tfdp2	mmu_circ_0016010	−78.0619	0.012609
chr14_13995006_14053023_+	Atxn7	mmu_circ_0005063	−76.833	0.011972
chrX_167363544_167374186_−	Prps2	NA	−75.4238	0.013103
chr4_150431522_150431985_+	Rere	mmu_circ_0011440	−65.5894	0.012612
chr14_29333708_29396583_−	Cacna2d3	NA	−65.0899	0.013294
chr6_51562017_51589065_+	Snx10	NA	−64.6467	0.014026
chr14_62733627_62743998_−	Ints6	NA	−64.3073	0.01361
chr7_56982624_56985163_−	Gabrg3	mmu_circ_0014270	−62.3892	0.014665
chr13_91968946_91972285_−	Rasgrf2	mmu_circ_0004873	−61.1195	0.015346
chr14_79369077_79370097_−	Naa16	mmu_circ_0005462	−58.7143	0.014048
chr5_35935551_35936132_+	Afap1	mmu_circ_0012596	−58.398	0.005375
chr15_25737449_25742449_+	Myo10	mmu_circ_0005560	−58.3059	0.014391
chr6_126013159_126015695_+	Ano2	NA	−56.5138	0.01669
chr7_91449753_91872500_+	Dlg2	NA	−55.3916	0.016159
chr5_21194399_21207641_+	Gsap	NA	−54.8773	0.01561
chr8_25592451_25594166_−	Letm2	NA	−54.5229	0.01658
chr2_44570418_44591990_−	Gtdc1	NA	−51.9963	0.007337
chr5_128761339_128780376_−	Rimbp2	mmu_circ_0012149	−51.8159	0.016427
chr13_8672404_8701731_+	Adarb2	mmu_circ_0004840	−51.1778	0.007795
chr17_44717191_44724901_−	Runx2	mmu_circ_0000795	−50.8107	0.012892
chr18_34268297_34300061_+	Apc	mmu_circ_0007251	−45.3232	0.013193
chr3_118759655_118811267_+	Dpyd	NA	−44.9853	0.018425
chr11_117290397_117291036_+	9-Sep	mmu_circ_0002788	−44.5989	0.01282
chr14_33388799_33402923_−	Mapk8	NA	−43.5971	0.018773
chr9_121364000_121392051_+	Trak1	NA	−36.6467	0.007726
chr2_80520371_80525678_−	Nckap1	mmu_circ_0001039	−33.3722	0.010502
chr7_75140075_75149088_−	Sv2b	NA	−29.0699	0.002013
chr16_60424720_60425384_−	Epha6	mmu_circ_0000694	−29.0005	0.01947
chr4_59207760_59213977_+	Ugcg	NA	−28.8672	0.006424
chr17_74770242_74799411_+	Ttc27	NA	−23.7796	0.018256
chr12_66506105_66506822_−	Mdga2	mmu_circ_0004128	−20.9794	0.012785
chr7_133862102_133870087_+	Fank1	mmu_circ_0013965	−18.4343	0.011266
chr1_164340728_164347879_+	Nme7	mmu_circ_0008373	−18.2923	0.005958
chr2_115669581_115687169_+	BC052040	mmu_circ_0001057	−14.198	0.016445
chr5_122429485_122433537_+	Anapc7	mmu_circ_0012079	14.58085	0.012689
chr3_125914637_125915461_−	Ugt8a	mmu_circ_0010433	14.9599	0.014812
chr4_84390888_84414348_−	Bnc2	mmu_circ_0001224	19.49736	0.007617
chr5_139181416_139184719_+	Heatr2	mmu_circ_0012236	56.47568	0.006519
chr8_40348939_40359448_+	Micu3	NA	61.38255	0.014883
chr8_125886933_125888193_−	Pcnxl2	NA	62.23414	0.014888
chr17_6198671_6201834_+	Tulp4	NA	64.37413	0.003266
chr16_55918537_55923040_−	Cep97	NA	71.82017	0.013319
chr7_139845584_139852657_+	Gpr123	NA	74.60841	0.014702
chr13_59473687_59482604_−	Agtpbp1	mmu_circ_0004710	78.31382	0.012486
chr15_64191483_64349913_−	Asap1	NA	78.71352	0.01227
chr1_89627760_89671271_+	Agap1	NA	79.23549	0.014461
chr2_163040626_163042799_+	Ift52	NA	80.06381	0.012829
chr4_132910691_132913826_−	Fam76a	NA	82.11838	0.013523
chr16_96152970_96154195_+	Wrb	NA	91.7975	0.011942
chr17_50782260_50800195_−	Tbc1d5	NA	92.09871	0.012202
chr8_31849309_31968185_−	Nrg1	NA	92.93369	0.012086
chr13_119782771_119790287_−	Zfp131	NA	94.26963	0.011376
chr5_89038450_89058044_+	Slc4a4	NA	94.35752	0.012803
chr7_97636376_97653207_+	Rsf1	NA	95.61444	0.010142
chr7_37649685_37658660_−	Zfp536	NA	99.19821	0.011147
chr6_97189528_97199321_−	Uba3	mmu_circ_0013630	100.1458	0.009487
chr19_46449828_46453315_+	Sufu	NA	101.4795	0.009533
chr5_76265790_76274043_−	Clock	mmu_circ_0012787	102.1796	0.011921
chr5_34419997_34444913_+	Fam193a	mmu_circ_0001340	103.6374	0.009949
chr4_59596862_59601567_+	Hsdl2	NA	105.3806	0.010751
chr6_119369859_119370408_−	Adipor2	mmu_circ_0013007	107.8557	0.009198
chr10_30762739_30771840_−	Ncoa7	mmu_circ_0002155	108.1671	0.008979
chr8_79070370_79076538_+	Zfp827	mmu_circ_0015001	111.6757	0.008381
chr14_99179314_99196451_+	Pibf1	NA	112.7662	0.010379
chr2_10393264_10461472_+	Sfmbt2	NA	114.4586	0.008053
chr4_102487210_102515611_+	Pde4b	NA	114.7701	0.007858
chr16_62769606_62776383_−	Nsun3	NA	114.8451	0.009533
chr7_126886207_126889060_–	Tmem219	NA	115.4738	0.008434
chr1_139039999_139110485_+	Dennd1b	mmu_circ_0000082	116.1391	0.007941
chr1_156620838_156625583_+	Abl2	NA	116.4107	0.007942
chr4_74357341_74377800_+	Kdm4c	NA	116.9301	0.007803
chr4_150468694_150470391_+	Rere	NA	120.5443	0.00708
chr3_152317613_152320743_–	Fam73a	NA	122.1839	0.007135
chr11_102860001_102865660_−	Eftud2	mmu_circ_0002659	122.9623	0.008327
chr12_55757925_55762737_–	Ralgapa1	mmu_circ_0004092	124.9689	0.006679
chr3_131599812_131607463_+	Papss1	NA	125.3036	0.00663
chr13_94210576_94233180_−	Scamp1	NA	139.1756	0.005192
chr10_49522142_49535499_−	Grik2	NA	148.6745	0.004614
chr11_23281305_23282726_+	Xpo1	mmu_circ_0002894	153.5301	0.004153
chr5_44592732_44799437_−	Ldb2	NA	163.2662	0.003521
chr18_82955523_82957449_+	Zfp516	mmu_circ_0000909	178.6214	0.00208
chr8_88156461_88158265_+	Heatr3	mmu_circ_0015094	451.1435	2.85E-06

### Isolation and sequencing of RNA

Total RNA was isolated using QIAzol™ (Qiagen, Germany) and miRNeasy™ kits (Qiagen, Germany), including additional DNase I digestion. Then, ribosomal RNA was removed using the Ribo-Zero™ Magnetic Gold kit (Qiagen, Germany), and enzymatic degradation of linear RNA was done using Rnase R enzyme. Fragmentation buffer was added to fragments. The first chain of complementary (c)DNA was synthesized with six-base random hexamers. Then, the buffer, dNTPs, RNase H, and DNA polymerase I were added to synthesize the second chain of cDNA. Purification using the QiaQuick™ PCR kit and elution using EB buffer after terminal repair, processing of base A, and addition of sequencing joints were carried out. For next-generation sequencing, 0.5 μg of ribosomal RNA-depleted RNA was fragmented and primed. Sequencing libraries were constructed using TruSeq™ RNA Sample Preparation kits (Illumina, San Diego, CA, USA) and were sequenced by Illumina HiSeq™ 2500 flow cells.

### Computational analysis of circRNAs

First, reads were mapped to the latest University of California Santa Cruz transcript set using Bowtie2 v2.1.0 (http://bowtie-bio.sourceforge.net/bowtie2/index.shtml). Gene expression was estimated using RSEM v1.2.15. For analyses of lincRNA expression, we used the transcripts set from Lncipedia (www.lncipedia.org/). Trimmed mean of M-values (TMM) was used to normalize gene expression. Differentially expressed genes were identified using the edgeR program. Genes showing altered expression with *p* < 0.05 and >1.5-fold changes were considered to have differential expression. Pathway analysis and network analysis were undertaken using Ingenuity (IPA). IPA computes a score for each network according to the fit of a set of supplied focus genes. These scores indicate the likelihood of focus genes belonging to a network *versus* those obtained by chance. Score >2 indicates a ≤99% confidence that a focus gene network was not generated by chance alone. The canonical pathways generated by IPA are the most significant for the uploaded dataset. Fischer’s exact test with a false discovery rate (FDR) option was used to calculate the significance of the canonical pathway.

For analyses of circRNA expression, reads were to mapped a genome using STAR. DCC was employed to identify and estimate expression of a particular circRNA. TMM was used to normalize gene expression. Differentially expressed genes were identified using edgeR. miRanda was employed to predict the miRNA target of the circRNA. R (R Institute for Statistical Computing, Vienna, Austria) was used to generate figures.

### circRNA verification by real-time reverse transcription-quantitative polymerase chain reaction (RT-qPCR)

We wished to validate the reliability of high-throughput RNA-sequencing (RNA-seq) and to explore expression of circRNAs during aging. Hence, expression of circRNAs was measured by RT-qPCR. With reference to the method described by Memczak, two sets of primers for each circRNA were designed using Primer Express v5.0 (Table 2): an outward-facing set to amplify only the circRNA, and an opposite-directed set to amplify the linear form.

**Table 2:** PCR primers.

Primer name	Sequence (5′->3′)	Product size (bp)
mmu_circ_0012931	circ_0012931-F1: TCCACTTGTTAAGATACCTC circ_0012931-R1: GCAAGAGTAGATACATAATTCC	168
β-actin	β-actin-F1: GCTTCTAGGCGGACTGTTAC β-actin-R1: CCATGCCAATGTTGTCTCTT	100
rno_circ_NF1-419	rno_circ_NF1-419-F1: AGTCGAATTTCTACAAGCTTC rno_circ_ NF1-419-R2: AGCTTCTCCAAATATCCTCAT	179

Total RNA was extracted (TRIzol® Reagent, Life Technologies, Carlsbad, CA, USA), digested using RNase R, and purified. cDNA was synthesized using the Geneseed^®^ II First Strand cDNA Synthesis kit (Agilent Technologies, Santa Clara, CA, USA). Outward-facing primers were designed to amplify the fragment across the junction from cDNA, then the fragment was sequenced by Sangon Biological Engineering (Shanghai China). RT-qPCR was undertaken using Geneseed^®^ qPCR SYBR^®^ Green Master Mix (Agilent Technologies). PCR-specific amplification was conducted with an ABI 7500 system (Applied Biosystems, Foster City, CA, USA). Expression of circRNAs was defined based on the threshold cycle (Ct), and relative expression was calculated *via* the 2^−ΔΔCt^ method. Glyceraldehyde 3-phosphate dehydrogenase served as an IS control with all reactions done in triplicate.

### Western blotting

Briefly, global brain tissue was dissected from treated mice (purchased from Beijing HFK Bioscience; SCXK (Jing) 2014-0004) and proteins extracted with a radioimmunoprecipitation assay lysis buffer (T-PER^™^ Tissue Protein Extraction Reagent; catalog number, 78510; Thermo Scientific, Waltham, MA, USA). Proteins were separated by sodium dodecyl sulfate–polyacrylamide gel electrophoresis and transferred onto polyvinylidene fluoride (PVDF) membranes. After blockade with 5% nonfat dry milk in Tris-buffered saline (20 mM Tris-HCl, 500 mM NaCl, pH 7.4) with 0.2% Tween-20 (T104863; Aladdin, Beijing, China), the PVDF membranes were probed with antibodies overnight at 4 °C, followed by incubation with a horseradish peroxidase-conjugated goat anti-mouse (G2211-1-A; Servicebio, Beijing, China) or goat anti-rabbit (G2210-2-A; Servicebio) IgG secondary antibody (1:2000 dilution). Band intensity was quantified using ImageJ (National Institutes of Health, Bethesda, MD, USA).

### Statistical analyses

Data are the mean ± SD of at least three independent experiments. Significant differences between treatments were analyzed by one-way analysis of variance (ANOVA) at *p* < 0.05 using SPSS (IBM, Armonk, NY, USA) and Prism 5 (GraphPad, San Diego, CA, USA).

## Results

### The HSHF diet disrupts the GM

**Fig. 1:**
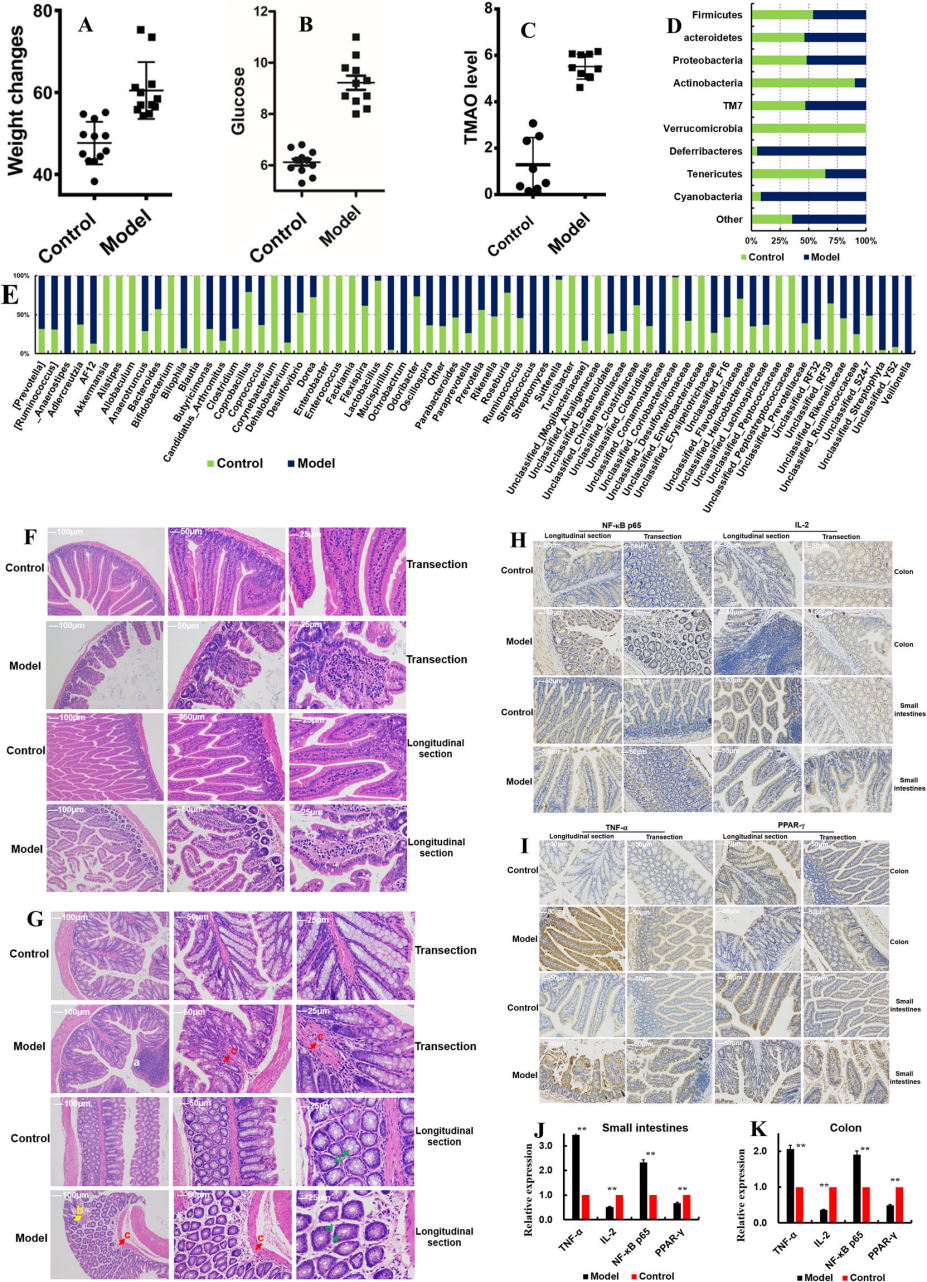
Dysbacteriosis affects the homeostatic balance of the intestine in mice fed a diet high in sugar and fat for 4 months. **A** Influences of a diet high in sugar and fat on the bodyweight of mice. **B** Influences of a diet high in sugar and fat on the blood glucose level of mice. **C** Influences of a diet high in sugar and fat on TMAO levels in serum. **D** Influences of a diet high in sugar and fat on the gut microbiota at the phylum level. **E** Influences of a diet high in sugar and fat on the gut microbiota at the genus level, see Fig. S2. **F**, **G** Influences of a diet high in sugar and fat on intestine (**F**) and colon (**G**) histopathology using H&E staining, see the pathologic observations in other organs (livers, renal, spinal marrow, spleen, adipose and heart) in Fig. S2. **H**–**K** Influences of a diet high in sugar and fat on intestine and colon histopathology using immunohistochemical analyses. Data are the mean ± SD of more than 8 independent experiments (the histopathology data were the mean ± SD of more than 3 independent experiments). *p < 0.05 and **p < 0.01 vs. the model group by one-way ANOVA, followed by the Holm–Sidak test.

**Fig. 2:**
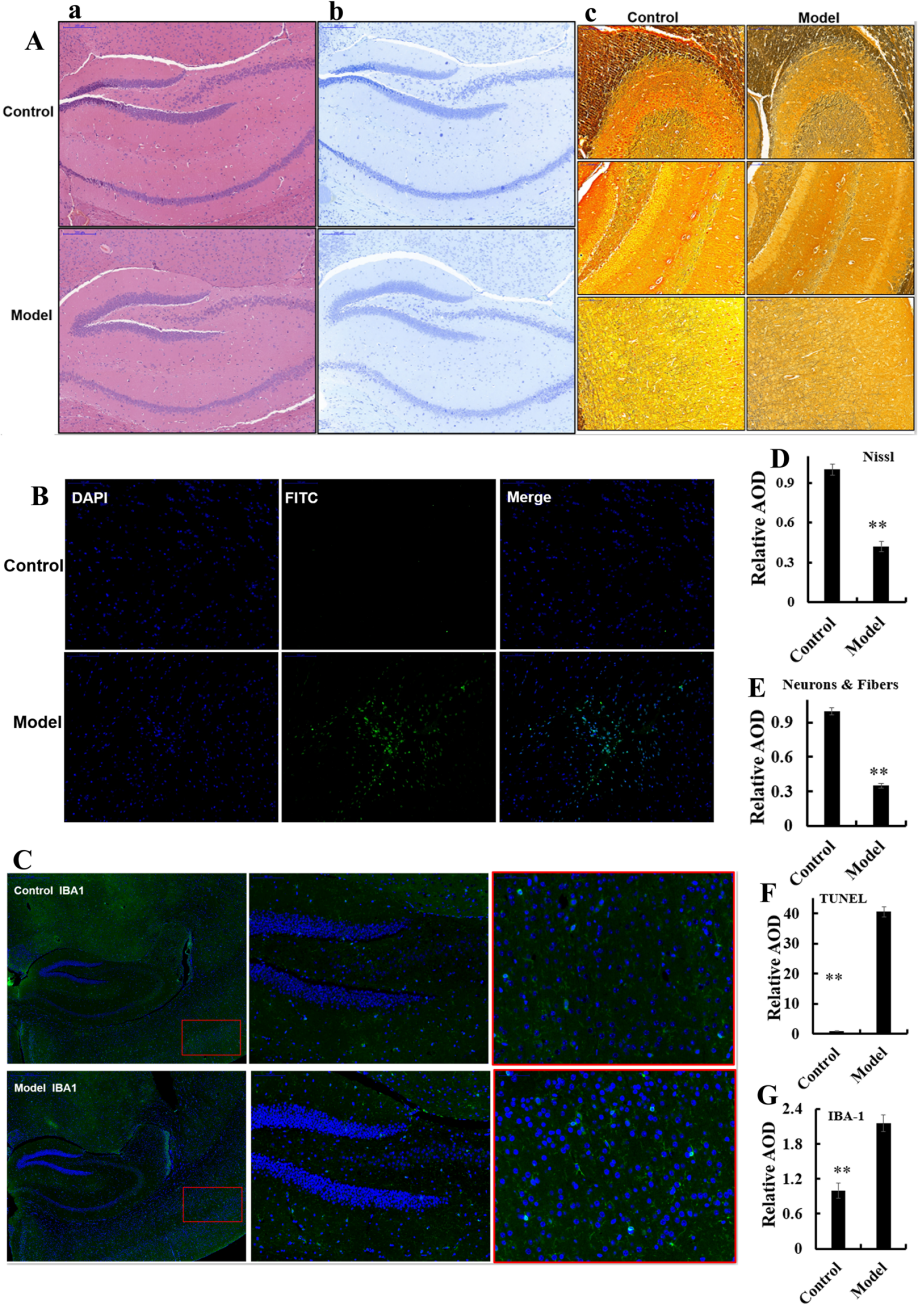
Dysbacteriosis affects brain histopathology in mice fed a diet high in sugar and fat for 4 months. **A** Samples were stained using hematoxylin and eosin (H&E), see also Fig. S3A-a. **B**, **D** Samples underwent Nissl staining, see also Fig. S31A-b. **C**, **E** Samples were stained using silver, see also Fig. S3A-c. **D**, **F** Samples underwent TUNEL staining, see also Fig. S3A-d. **C**, **G** Samples were stained using immunofluorescent microglial of IBA-1, see also Fig. S3B-b. Data are the mean ± SD of 5 independent experiments. *p < 0.05 and **p < 0.01 vs. the model group by one-way ANOVA, followed by the Holm–Sidak test.

The bodyweights of mice fed a HSHF diet were higher than those fed a standard diet (control, *p* < 0.05) (Fig. 1A); levels of blood glucose (Fig. 1B) and TMAO (Fig. 1C) were also higher (*p* < 0.05). These data suggested that the HSHF diet utilized caused increases in bodyweight, blood glucose level, and TMAO level. Sequencing of the 16 S rRNA gene showed that the HSHF diet significantly decreased the operational taxonomic units (OTUs) of bacteria of the phyla Acidobacteria, Verrucomicrobia, Tenericutes, and Firmicutes, while increasing the OTUs of bacteria of the phyla Bacteroidetes, Proteobacteria, Deferribacteres, Cyanobacteria and Actinobacteria (*p* < 0.05) (Fig. 1D and Fig. S1A). The OTUs of bacteria from the genera *Bifidobacterium* (213.12-fold change compared with that in the control group), *Coriobacteriaceae* (51.02), *Sutterella* (18.84), *Lactobacillus* (14.46), *Coprobacillus* (3.86), *Roseburia* (3.56), *Odoribaccter* (2.76), *Dorea* (2.65), and *Flavobacteriaceae* (2.39) were decreased significantly (*p* < 0.05) (Fig. 2E), whereas those of *AF12* ( − 6.64-fold changes), *Bliophila* (−13.37), *Butyricimonas* (−2.14), *Paraprevotella* (−2.76), *Bacteroidales* (−2.85), *Streptophyta* (−20.66), *Mucispirillum* (−18.50), *Candidatus Arthromitus* (−5.01), [*Mogibacteriaceae*] (−4.92), *Dehalobacterium* (−5.87), *RF32* (−4.46), *Anaerotruncus* (−2.43), *Erysipelotrichaceae* (−2.73), and *Ruminococcaceae* (−2.98) were increased significantly (*p* < 0.05) (Fig. 1E and Fig. S1B) following administration of the HSHF diet. The whole GM was different between the two groups of mice: control mice and those fed a HSHF diet. Principal component analysis (Fig. S1C), Venn diagram (Fig. S1D), and linear discriminant analysis effect size (LEfSe) analysis (Fig. S1E and S1F) could be used to distinguish between these two groups. Hence, the HSHF diet could induce dysbacteriosis in mice. These results are almost identical to data from studies showing that intestinal dysbacteriosis interacts with fat^21^.

### The HSHF diet changes the steady state of the intestine

Intestinal dysbacteriosis were considered first affects the intestinal physiology^22^. The pathology of the small intestine and colon of mice fed the HSHF diet was different from that of mice fed the standard diet. Changes induced by the HSHF diet included: cell shrinkage; reduction of cell turnover; cytoplasmic vacuolar changes; blurred and indistinct cell boundaries; severe shedding of intestinal villi; scattering of damaged tissue blocks (Fig. 1F, G).

Immunohistochemical analyses showed that expression of some proinflammatory markers was affected. Expression of tumor necrosis factor (TNF)-α and nuclear factor-kappa B (NF-κB p65) was activated (*p* < 0.05) (Fig. 1H–K), whereas expression of interleukin (IL)−2 and peroxisome proliferator-activated receptor (PPAR)-γ was inhibited (*p* < 0.05) (Fig. 1H–K). RNA-seq of small-intestinal tissues revealed that expression of 42 RNAs was upregulated and that of 68 RNAs was downregulated (|log2FC| > 1.0, FDR < 0.05 vs. control) (Table S1, Fig. S2A). For example, expression of *Hspa1a*, *Hspa1b*, *P2ry4*, *Enpp7*, *Ano3*, *Hsph1*, *Nos1ap*, *Slc5a12*, *Slc5a4a*, *Gm5286*, *Paqr9*, *G0s2*, *Trim50*, *Lep*, *Apoa2*, *Retnlb*, *Gsdmc*, *TCONS_00019039*, *Fam205a4*, *Gm20708*, *Exosc6*, *Pde2a* and *Gm10184* mRNAs was changed, and these were all related to intestinal injury/leakage, inflammation, and immunity. Pathway analysis using the Kyoto Encyclopedia of Genes and Genomes (KEGG) database showed that the important enrichment pathways were the: “PPAR signaling pathway” (mRNA of *APOA2*, *Acaa1b*, *Cyp4a10*, *Fabp1*, *Hmgcs2*, *Me1*); “biosynthesis of unsaturated fatty acids”; “fructose and mannose metabolism”; “glycolysis/gluconeogenesis”; “fatty acid elongation”; “carbon metabolism”; “renin secretion”; “renin–angiotensin system”; “AMPK signaling pathway” (mRNA of *G6pc*, *Fbp1*, *Srebf1*, *Lep*). Together these results demonstrated that dysbacteriosis influenced intestinal injury/leakage, the inflammatory response, and energy metabolism. Furthermore, histopathology of the liver (Fig. S2B), kidney (Fig. S2E), spinal marrow (Fig. S2E), spleen (Fig. S2D), adipose (Fig. S2C) and heart (Fig. S2F) tissues showed that the HSHF diet not only induced dysbacteriosis, but also activated inflammation and damage to multiple organs.

### The HSHF diet influences the brain cholinergic system and inflammation

**Fig. 3:**
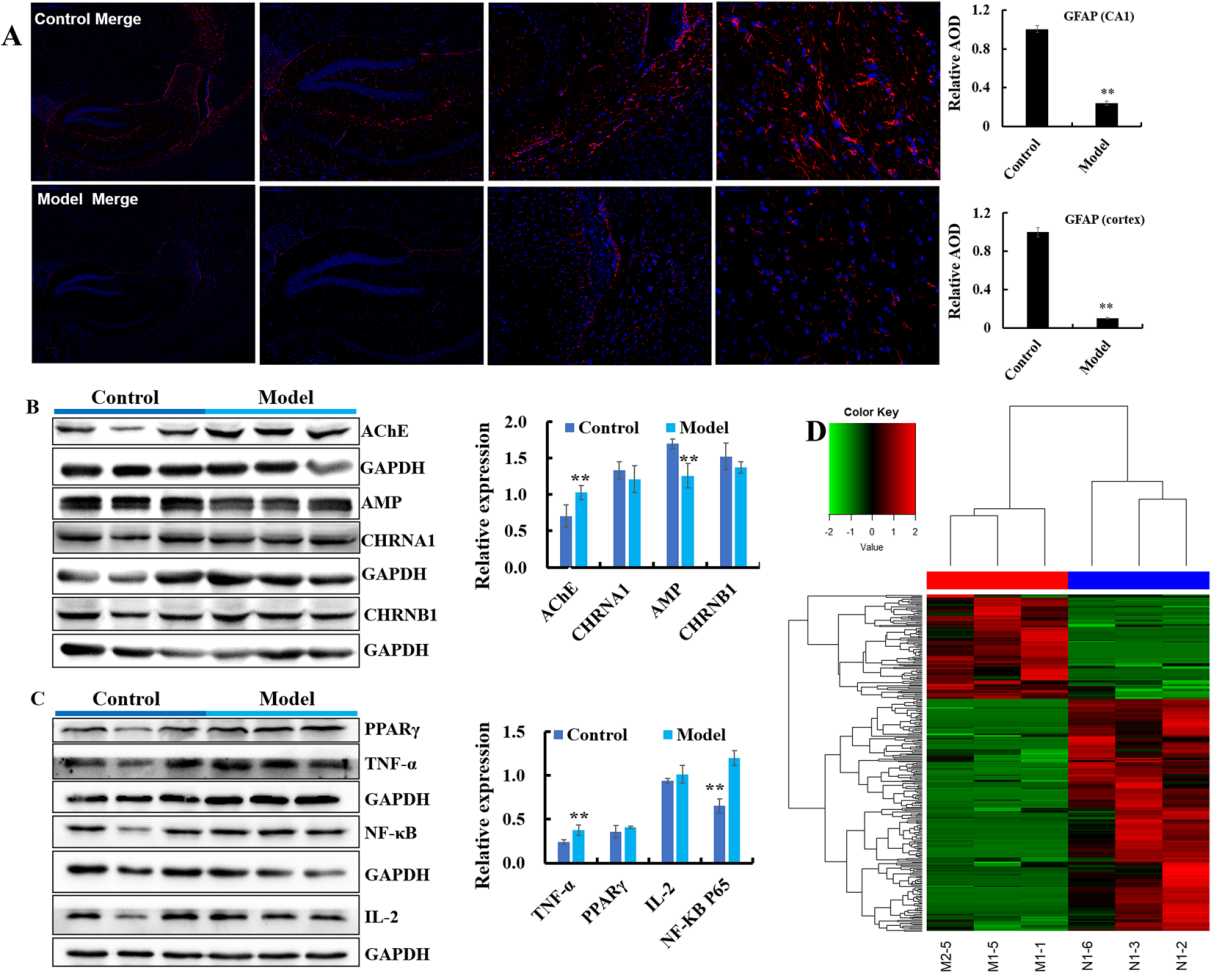
Dysbacteriosis affects brain functions and circRNA sequencing in mice fed a diet high in sugar and fat for 4 months. **A** Samples were stained using an immunofluorescent antibody of GFAP, see also Fig. S3B-a. **B**, **C** Dysbacteriosis implicated the cholinergic system, inflammation and immune system in the brain. **D** Heatmap showing different expression of circRNAs in brain samples from mice fed a diet high in sugar, see Table 1. Data are the mean ± SD of 3 independent experiments. *p < 0.05 and **p < 0.01 vs. the model group by one-way ANOVA, followed by the Holm–Sidak test.

The HSHF diet was administered to mice to disturb the GM. Brain sections from mice were analyzed using various stains. H&E (Fig. 2Aa, Fig. S3A–A), Nissl (Fig. 2Ab, D and Fig. S3A–B), silver (Fig. 2Ac, E and Fig. S3A–C) and TUNEL (Fig. 2B–F and Fig. S3A–D) staining showed obvious changes in pathology in mice with HSHF diet-induced dysbacteriosis: reduction in the size and turnover of neurons; cytoplasmic vacuolar changes; nerve-fiber reduction. In particular, the apoptotic percentage in the hypothalamus was increased in mice suffering from dysbacteriosis, which suggested that the appetite of HSHF-diet mice increased and bodyweight soared, as seen previously^23^. Immunofluorescent staining of microglia and astrocytes using antibodies to IBA-1 (Fig. 2C, G, Fig. S3B–B) and GFAP (Fig. 3A, Fig. S3B–A) showed that the number of astrocytes was reduced significantly and microglial cells were activated in mice suffering from dysbacteriosis. Expression of the cholinergic neuron of AChE (Acetylcholinesterase) (Fig. 3B, *p* < 0.05), Amphiphysin (AMP) (Fig. 3B, *p* < 0.05), Acetylcholine receptor of CHRNB1 and CHRNA1 measurement showed that the cholinergic system was affected. Expression of TNF-α (*p* < 0.05) (Fig. 3C) and NF-κB p65 (*p* < 0.05) (Fig. 3C) was activated, whereas expression of PPAR-γ and IL-2 showed no obvious differences (*p* > 0.05) (Fig. 3C). These data demonstrated that atrophy, inflammation, or the immune response were imbalanced in the brains of mice with HSHF diet-induced dysbacteriosis.

### The HSHF diet accelerates circRNAs degradation in mice brains

A total of 7362 circRNAs were identified in the brains of mice fed a HSHF diet (Fig. 3). Among them, 287 differentially expressed circRNAs were in the control group *vs*. model group (fold change >1.50, *p* < 0.05) (Fig. 3D, and Table 1, circBase): expression of 88 circRNAs was upregulated, and expression of 199 circRNAs was downregulated. This finding indicated that the circRNAs had different expression profiles in slender and obese mice. Bioinformatics analysis of differentially expressed circRNAs showed that dysbacteriosis could influence the brain in terms of metabolism, synaptic transmission and plasticity, and endogenous hormone levels. These actions would affect the activities and functions of the brain, but also affect heart function (e.g., circadian rhythm, dilated cardiomyopathy, and arrhythmogenic right ventricular cardiomyopathy). These data demonstrated that dysbacteriosis could affect brain circRNA-seq directly or indirectly.

### Networks interaction of the GM, neurotransmitters, intestinal mRNAs, and brain circRNAs

#### Network interaction of the GM and intestinal neurotransmitters

**Fig. 4:**
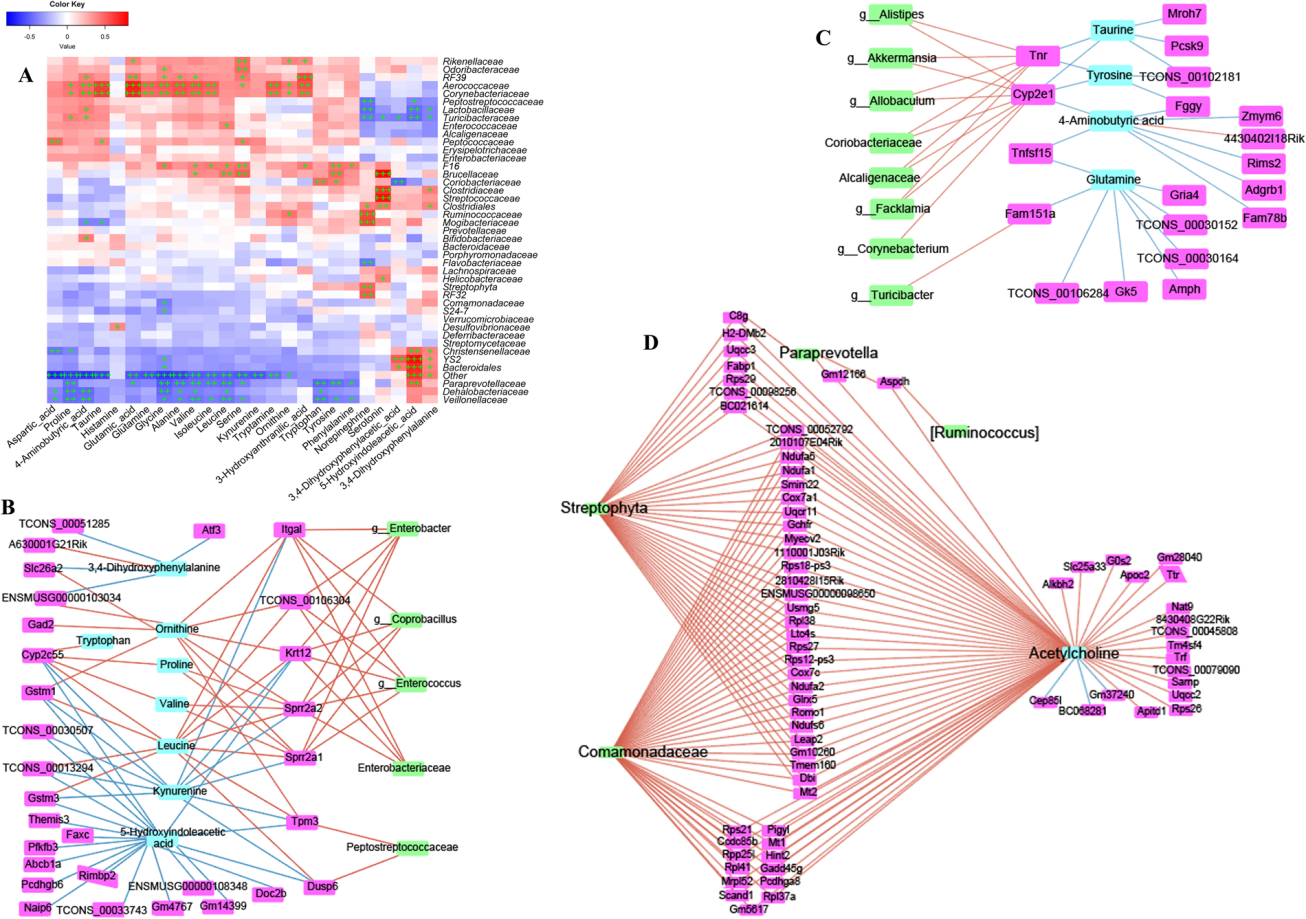
Dysbacteriosis affects neurotransmitter metabolism by targeting the gut–brain axis in mice fed a diet high in sugar and fat for 4 months. **A** Combined data analyses between the gut microbiota (genera) and neurotransmitters in intestine tissues. **B**–**D** Combined data analyses between the gut microbiota (genera), transcriptome (mRNA) and neurotransmitters in intestine tissues. Data are the mean ±SD of more than 8 independent experiments.

To identify the influences of the GM on neurotransmitters in the intestine, combined data analyses of the GM and neurotransmitters in fimo were done (*R*^*2*^ > 0.85). Some members of *Aerococcaceae*, *Corynebacteriaceae*, *Brucellaceae*, *F16*, *Paraprevotellaceae*, *Veillonellaceae*, and *Dehalobacteriaceae* were the key bacteria related to the production and/or secretion of neurotransmitters (Fig. 4A). They influenced proline, glycine, leucine, and serine (Fig. 4A). Phenylalanine, tryptophan, and tyrosine were influenced by bacterial in *Veillonellaceae*, *Dehalobacteriaceae*, *Paraprevotellaceae*, *Coriobacteriaceae*, *Brucellaceae,* and *F16*, but how these bacteria produce them, and then participate or intervene in host metabolic pathways, merits further study.

The norepinephrine level was influenced by bacteria from *Streptophyta*, *RF23, Flavobacteriaceae*, *Lactobacillaceae*, *Ruminococcaceae*, *Turicibacteraceae, Clostridiales, Mogibacteriaceae* and *Peptostreptococcaceae* (Fig. 4A). The 5-hydroxy tryptamine (5-HT) level was influenced by bacteria from *Streptococcaceae*, *Helicobacteraceae*, *Clostridiales, Turicibacteraceae, Clostridiaceae*, and *Brucellaceae* (Fig. 4A). The level of 5-Hydroxyindoleacetic acid (5-HIAA), the primary metabolite of 5-HT, was influenced by bacteria from *YS2*, *Lactobacillaceae*, *Turicibacteraceae*, *Christensenellaceae, Peptostreptococcaceae*, *Paraprevotellaceae,* and *Bacteroidales* (Fig. 4A).

Upon combination with data for intestinal mRNAs, we found that intestinal neurotransmitters also influenced intestinal genes, and then bacteria. As shown in Fig. 4B, leucine was positive to the mRNA of glutathione S-transferase Mu 3 (*Gstm3)*, integrin subunit alpha L (*Itgal*), *Krt12*, small proline rich protein 2 A 2 (*Sprr2a2*), small proline rich protein 2 A 1 (*Sprr2a1*), tropomyosin 3 (*Tpm3*), dual specificity phosphatase 6 (*Dusp6*), and then to bacteria from *Coprobacillus*, *Enterococcus*, *Enterobacter*, *Enterobacteriaceae*, and *Peptostreptococcaceae*. The gene of cytochrome P450 family 2 subfamily E member 1 (Cyp2e1) influenced taurine, tyrosine, and 4-Aminobutyric acid (Fig. 4C), and then became involved with the bacteria of *Akkermansia*, *Allobaculum*, *Coriobacteriaceae*, *Alcaligenaceae*, *Corynebacterium Alistipes,* and *Facklamia* (Fig. 4C). Tenascin R was involved with several types of bacteria (Fig. 4C).

#### Network interaction of the GM and brain neurotransmitters

**Fig. 5:**
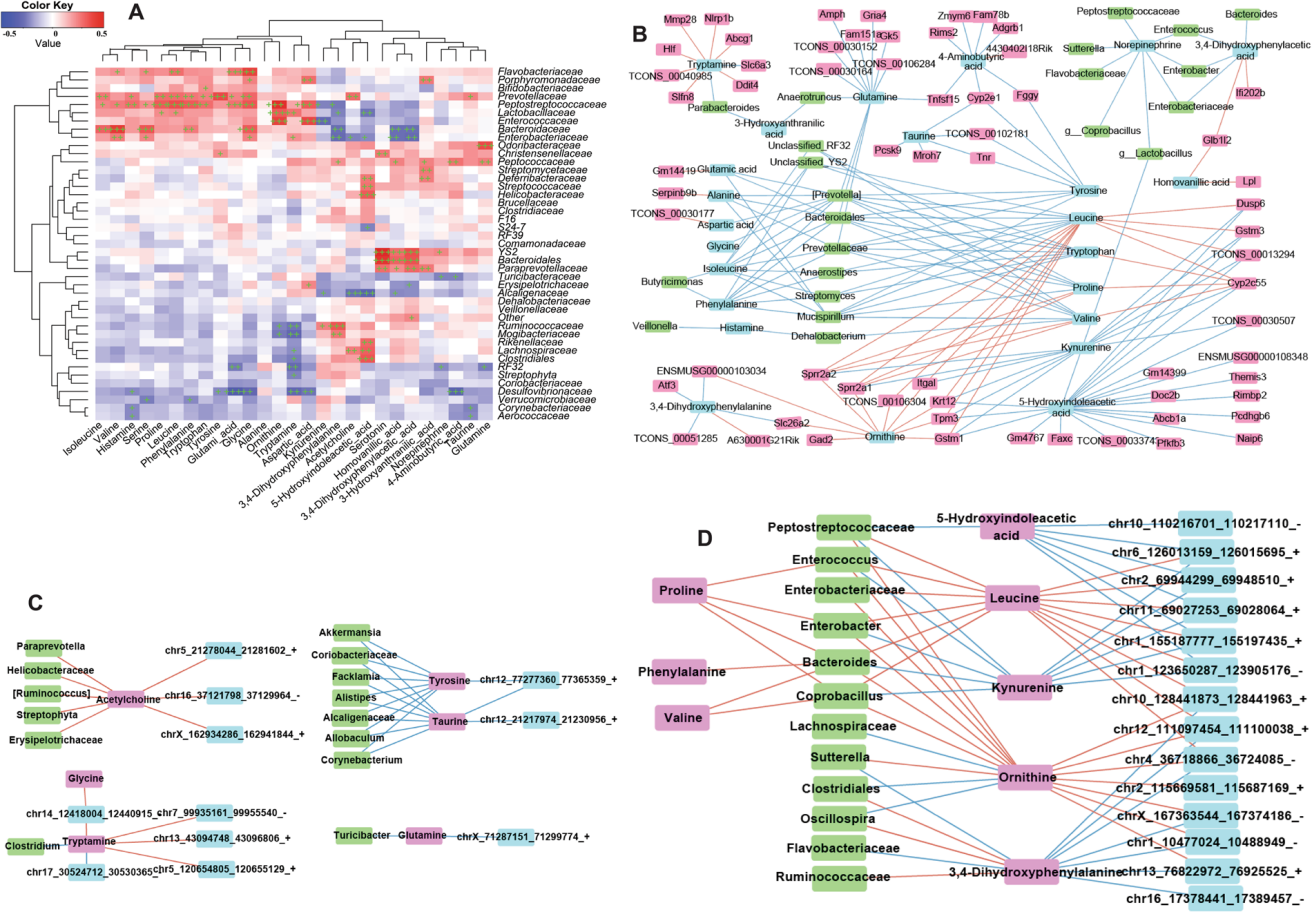
Dysbacteriosis affects neurotransmitter metabolism by targeting the gut–brain axis in mice fed a diet high in sugar and fat for 4 months. **A** Combined data analyses between the gut microbiota (genera) and neurotransmitters in brain tissues. **B** Combined data analyses between the gut microbiota (genera), transcriptome in the intestine, and neurotransmitters in brain tissues. **C**, **D** Combined data analyses between the gut microbiota (genera), neurotransmitters, and circRNAs in brain tissues. Data are the mean ± SD of more than eight independent experiments.

The bacteria of *Flavobacteriaceae*, *Porphyromonadaceae*, *Bifidobacteriaceae*, *Bacteroidaceae*, *Desulfovibrionaceae Enterococcaceae*, *Prevotellaceae*, *Lactobacillaceae* and *Peptostreptococcaceae* could interact with most brain neurotransmitters: isoleucine, valine, histamine, serine, proline, leucine, phenylalanine, tryptophan, tyrosine, glutamic acid, 3,4-Dihydroxyphenylalanine, kynurenine, 5-Hydroxyindoleacetic acid, tryptamine, glycine, and ornithine (Fig. 5A). The brain neurotransmitters homovanillic acid and 3,4-Dihydroxyphenylacetic acid were influenced by bacteria from *Bacteroidales*, *YS2*, *Paraprevotellaceae*, *Enterobacteriaceae* and *Bacteroidaceae* (Fig. 5A).

The “cholinergic hypothesis” has an important role in AD therapy^24^. The degenerative dysfunction of cholinergic neurons is a partial cause of memory deficit in dementia patients. The neurotransmitter acetylcholine (ACh) in the brain was implicated with several intestinal genes (Fig. 4D, Table S1 and enrichment analysis using the KEGG database), and might be intervened and/or promoted by bacteria from *Paraprevotella*, *[Ruminococcus]*, *Streptophyta* and *Comamonadaceae* (Fig. 4D). These data indicated that the GM has complex interactions with the host cholinergic system. We discovered that the norepinephrine level in the brain could: (i) influence bacteria of *Peptostreptococcaceae*, *Sutterella*, *Flavobacteriaceae* and *Coprobacillus*; interact with bacteria of *Enterococcus*, *Enterobacteriaceae*, and *Enterobacter*, and then interact with 3,4-dihydroxyphenylacetic acid; interact with bacteria from *Lactobacillus*, and then influence 5-hydroxyindoleacetic acid (Fig. 5B).

Glutamine, isoleucine, phenylalanine, tyrosine, leucine, tryptophan, proline and valine did not interact with a single type of bacteria (Fig. 5B). Hence, the interactions between GM, neurotransmitters, and host gene are complicated. More other complex interactions between the GM, mRNA-seq of intestinal tissue, and neurotransmitters of brain tissues are shown in Figs. 4 and 5. The neurotransmitters ACh, 4-aminobutyric acid, 5-hydroxyindoleacetic acid (5-HIAA), glutamine, serotonin, leucine, and kynurenine may also have important roles in the brain–GM axis, or the GM may be indispensable for the synthesis and secretion of neurotransmitters: the exact mechanism needs much more work.

#### Network interaction of the GM, brain neurotransmitters, and circRNAs

Next, the interactions of the GM, neurotransmitters, and circRNAs in brain tissues were analyzed. The ACh level in brain tissues was important for the interactions between the brain circRNA of chr5_21278044/21281602_+, chrX_162934286/162941844_+ and chr16_37121798/ 37129964_−, and bacteria from *Paraprevotella, Helicobacteraceae, Erysipelotrichaceae, [Ruminococcus]* and *Streptophyta* (Fig. 5C). The neurotransmitters 5-HIAA, leucine, 3,4-dihydroxyphenylacetic acid, kynurenine, ornithine, tryptamine, ACh, and tyrosine were the main metabolites influencing the GM and circRNAs (Fig. 5D), or they could influence multiple microorganisms and circRNAs (Fig. 5). Much more experimental evidence is needed to explain these associations.

5-HIAA is the main metabolite of 5-HT. A recent study showed that 5-HT directly stimulates and inhibits the growth of commensal bacteria in vitro, and exhibits concentration-dependent and species-specific effects^25^. Some bacteria may have important roles in the conversion of 5-HT to 5-HIAA. Bacteria from the family *Peptostreptococcaceae* could affect the mRNA of *Tpm3* and *Dusp6* (Figs. 4 and 5). Research has revealed a genetic proof-of-function for choline acetyltransferase (ChAT) in T cells during viral infection and identified a pathway of T-cell migration that sustains antiviral immunity^26^. Levels of ACh and ChAT were influenced primarily by bacteria from *Helicobacteraceae*, *Paraprevotella*, *[Ruminococcus]*, *Erysipelotrichaceae*, *Comamonadaceae,* and *Streptophyta* (Figs. 4 and 5). The role of the GM in TMAO byproducts from choline has been demonstrated^27^, and TMAO has been shown to be harmful in several diseases^28^. Therefore, the GM acts as a bridge to contribute to host nutrition/health by regulating the metabolism. The above-mentioned data, together with data from other studies, suggest that gut dysbacteriosis could impact circRNA sequences in the brain.

### TMAO byproducts influence circRNA levels in BV2 cells

**Fig. 6:**
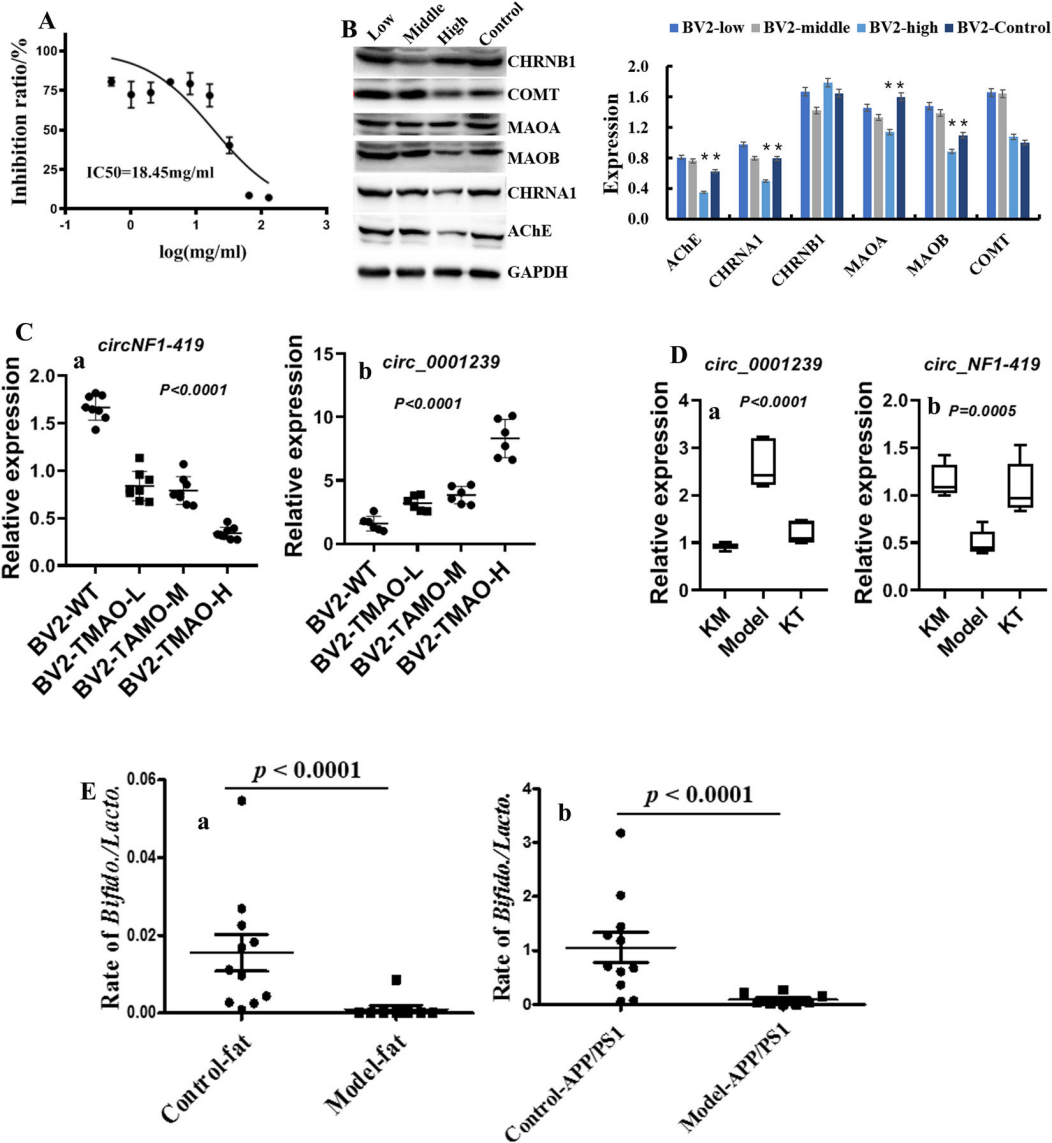
Effects of TMAO on BV2 cells. **A** Effects of TMAO on proliferation of BV2 cells, measured using the MTT assay. **B** Expression of MAOA, MAOB, COMT, AChE, AMP, CHRNA1, and CHRNB1 in TMAO- damaged BV2 cells. **C** Expression of *circNF1-419* and *circ_0001239* in TMAO- damaged BV2 cells. **D** Expression of *circNF1-419* and *circ_0001239* in *Kombucha tea-*treated mice. **E** Ratio of abundance of *Bifidobacterium* species and *Lactobacillus* species was imbalanced in mice fed a HSHF diet and in AD-like mice. Data are the mean ± SD of more than five independent experiments (and three independent experiments when using western blotting). *p < 0.05 and **p < 0.01 vs. the model group by one-way ANOVA, followed by the Holm–Sidak test.

In this additional experiment, expression of some circRNAs in a murine microglial cell line (BV2) was monitored after incubation with TMAO (18.45 mg/mL) (*p* < 0.05) (Fig. 6A). Expression of MAOA (Amine oxidase [flavin-containing] A), MAOB, COMT (Catechol O-methyltransferase), AChE, AMP, CHRNA1 and CHRNB1 was influenced by this concentration of TMAO (*p* < 0.05) (Fig. 6B), but a low concentration of TMAO did not cause obvious damage to BV2 cells. Percentage inhibition in BV2 cells did not differ at a TMAO concentration <16 mg/mL (Fig. 6A). Expression of some circRNAs, including *circNF1-419* and *circ_0001239*, was influenced by a low concentration of TMAO (*p* < 0.05) (Fig. 6C), indicating that some circRNAs might be sensitive responsive signaling molecules to TMAO. These data suggested that microbial metabolites might influence the formation and degradation of host circRNAs.

Levels of MAOA, MAOB, and COMT were affected after TMAO administration to BV2 cells (Fig. 6). Levels of some monoamine neurotransmitter were changed in mice fed the HSHF diet (Figs. 4 and 5). Taken together, these data suggested that behavior and stress would be influenced and, in these processes, the GM had an accelerant role. Therefore, the GM decides the transformation efficiency from choline to TMAO. The latter influences the levels of MAOA, MAOB, and COMT, which participate in the regulation of metabolism of neuroactive and vasoactive amines in the central nervous system (CNS) and peripheral tissues.

**Fig. 7:**
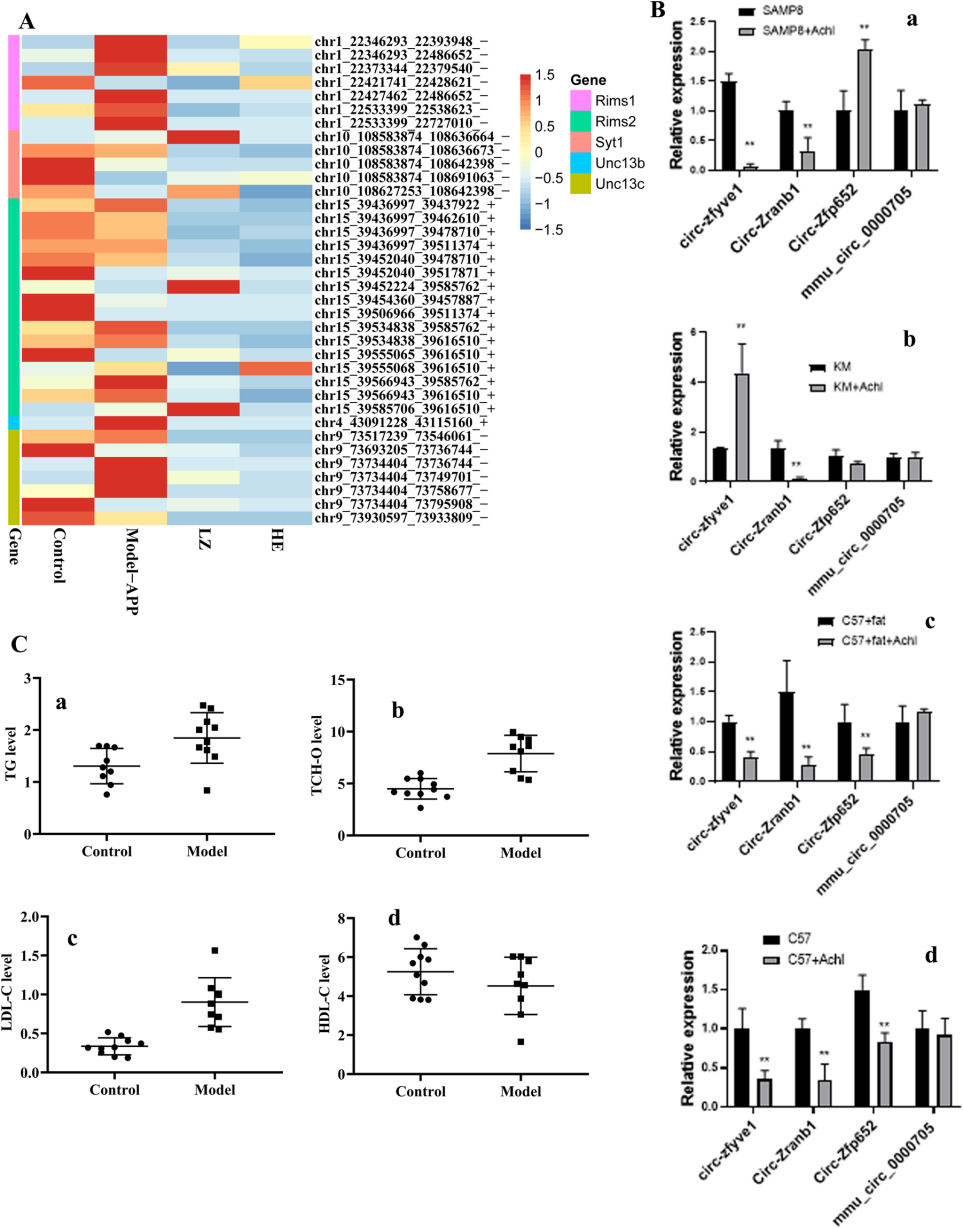
Effects of choline on circRNA expression and lipid metabolism. **A** Relative expression of circRNAs spliced from *Rims1/Syt1/Unc13b* enriched in the acetylcholine release cycle. **B** Expression of circRNAs in choline-treated mice using RT-qPCR. **C** Levels of total cholesterol (T-CHO), triglyceride (TG), low-density lipoprotein (LDL), and high-density lipoprotein (HDL) in mice fed a diet high in sugar and fat. Data are the mean ± SD of more than 6 independent experiments. *p < 0.05 and **p < 0.01 *vs*. the model group by one-way ANOVA, followed by the Holm–Sidak test.

circRNAs spliced from *Rims1/Syt1/Unc13b* could be enriched in the ACh release cycle (Fig. 7A). Levels of some of these circRNAs were measured in the brain tissues from different mice after choline treatment. Choline and/or its metabolite could change circRNA expression (Fig. 7B), which suggested that the GM also influences the absorption and conversion of choline. Pecoraro and colleagues suggested that a high-affinity PAC1 (Proteasome assembly chaperone 1) receptor presynaptically modulates hippocampal glutamatergic transmission acting through AChE in hippocampal CA1^29^, indicating that choline plays an important part in neurotransmitter homeostasis.

### The GM plays an important part in the conversion rate of choline to TMAO

The GM plays an important part in lipid homeostasis in blood, and in the causes and development of nervous-system diseases. In this study, the levels of total cholesterol (T-CHO, Fig. 7Ca), triglyceride (TG, Fig. 7Cb) and low-density lipoprotein (LDL, Fig. 7Cc) were also increased (*p* < 0.05), and the levels of serum TMAO (Fig. 3C), when compared to the HSHF-diet group (*p* < 0.05), and the high-density lipoprotein (HDL, Fig. 7Cd) were decreased (*p* < 0.05).

**Fig. 8:**
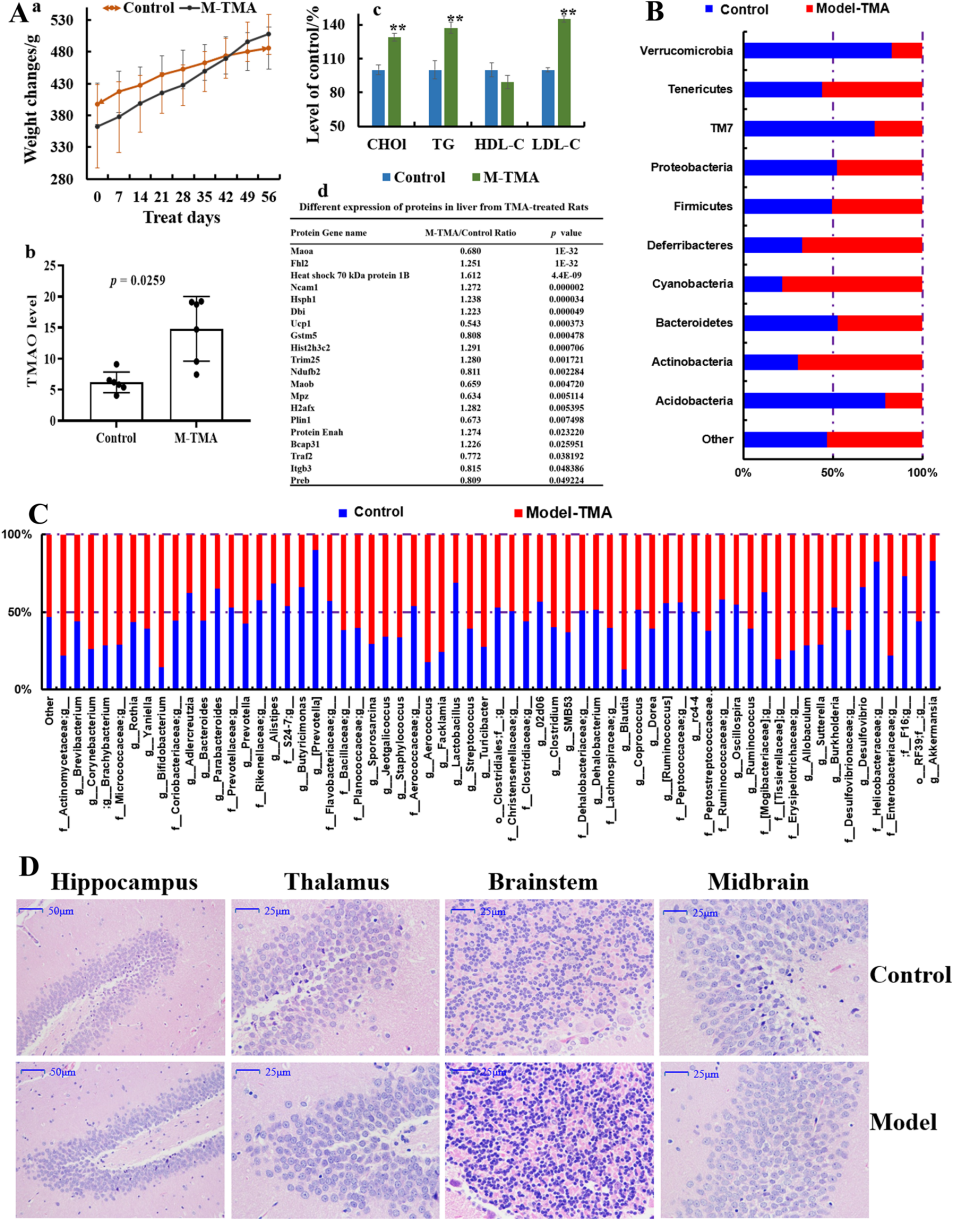
Effects of trimethylamine (TMA) on rats. **A** Effects on bodyweight changes (a), levels of T-CHO, LDL, HDL, and TG (b), levels of TMAO (c), and protein expression in liver tissues after intragastric administration of TMA at 0.2 mL/100 g/d of 2.5% for 6 weeks. **B** Effects on the gut microbiota at the phylum level, and (**C**) at genus level. **D** Pathologic changes in hippocampus, thalamus, brainstem, and midbrain after TMA administration. Data are the mean or mean ± SD of more than 8 independent experiments. *p < 0.05 and **p < 0.01 vs. the model group by one-way ANOVA, followed by the Holm–Sidak test.

Rats treated with TMA could put on weight continuously (Fig. 8Aa), had increased levels of TMAO in serum (Fig. 8Ab) and increased levels of total T-CHO (Fig. 8Ac), TG (Fig. 8Ac) and LDL (Fig. 8Ac) (*p* < 0.05) when compared with those in the model group (*p* < 0.05), whereas the HDL content was reduced (*p* < 0.05) (Fig. 8Ac). Data for sequencing of the 16 S rRNA gene showed that TMA decreased the OTUs of bacteria from Verrucomicrobia, TM7 and Acidobacteria (*p* < 0.05) (Fig. 8B), while increasing the OTUs for bacteria from Tenericutes, Deferribacteres, Actinobacteria, and cyanobacteria (*p* < 0.05) (Fig. 8B). At the genus level, the OTUs of bacteria from *Bifidobacterium*, *Aerococcus*, *Facklamia*, *Blautia* and *Enterobacteriace* were increased by TMA administration (*p* < 0.05) (Fig. 8C), whereas those of bacteria from *Prevotella*, *Lactobacillus*, *Helicobacteraceae* and *Akkermansia* were decreased significantly (*p* < 0.05) (Fig. 8C), which indicated that excessive TMA in the diet can induce dysbacteriosis. Histopathologic changes in the hippocampus, thalamus, brainstem, and midbrain of the TMA-induced group compared with those in the control group (Fig. 8D) included cell shrinkage, cytoplasmic vacuolar changes, blurred and indistinct cell boundaries, and scattered damaged tissue blocks. Also, the differential expression of proteins in the liver from TMA-treated rats revealed that TMA induced damage in the host. For example, the level of mitochondrial brown fat uncoupling protein 1 was decreased after treatment with excess TMA, together with a decrease in *Ndufb2* expression (Fig. 8Ad), which indicated that energy metabolism was imbalanced due to dysfunction of NADH dehydrogenase activity and oxidoreductase. Meanwhile, *Ncam1* expression was upregulated (Fig. 8Ad), suggesting that neurite outgrowth, synaptic plasticity, as well as learning and memory might have been influenced. Increased expression of heat shock 70 kDa protein 1B and B-cell receptor-associated protein 31 (Fig. 8Ad) indicated that the host stress response was activated and immune-system function might have been compromised. In agreement with results from other studies, TMA resulted in negative consequences for the host^29,30^ and, in the present study, TMA led to negative consequences for the nervous system.

### *C. albicans* and *K. pneumoniae* influence the cholinergic system in mice

**Fig. 9:**
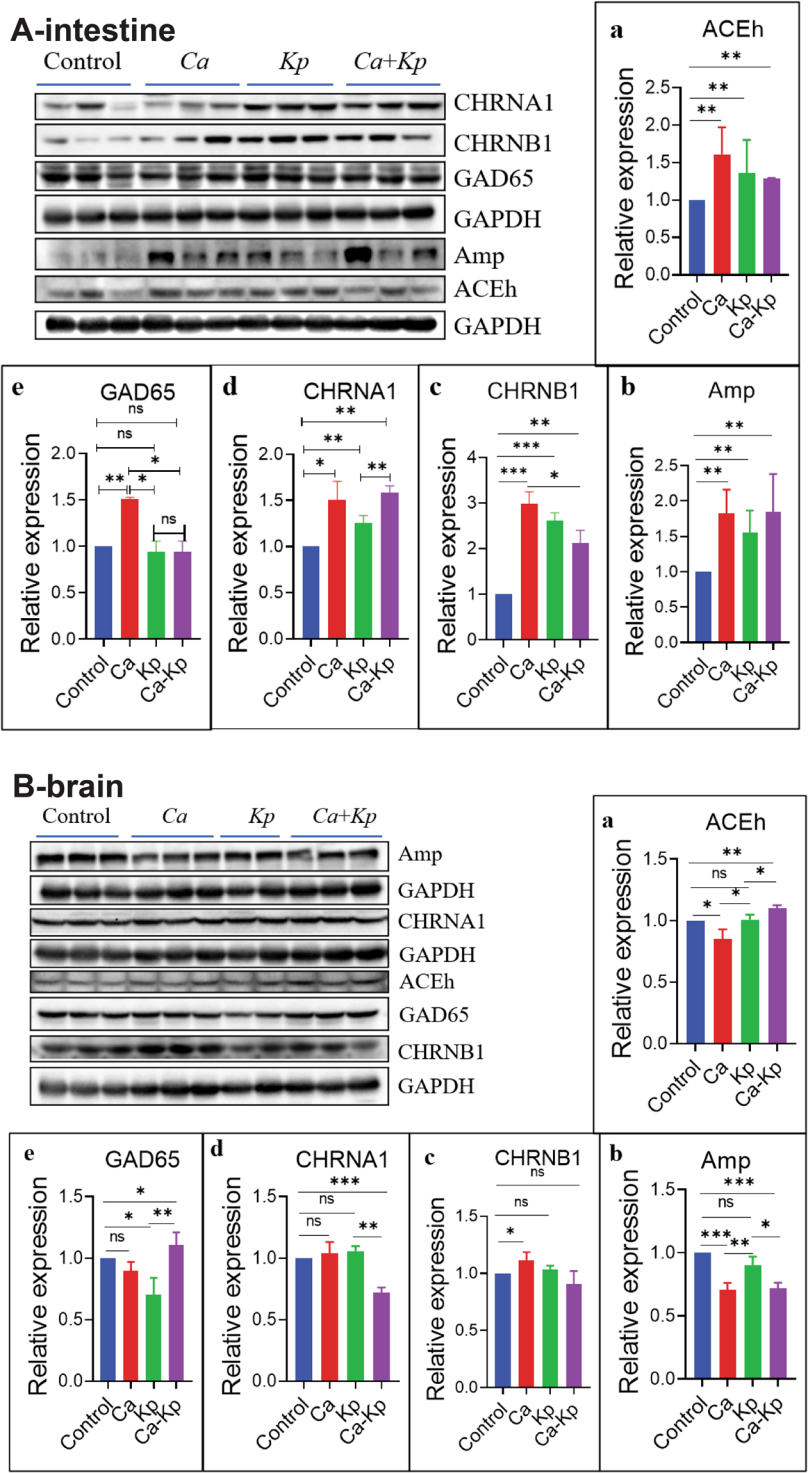
*C.albicans* and *Klebsiella pneumoniae* influence the cholinergic system in mice. **A** Expression of AChE, AMP, CHRNA1, CHRNB1, and GAD65 were changed in the intestine (**A**, *p* < 0.05) and brain (**B**). Data are the mean ± SD of three independent experiments. *p < 0.05 and **p < 0.01 vs. the model group by one-way ANOVA, followed by the Holm–Sidak test.

We wished to ascertain the potential routes of communication/interaction between the host and its resident bacteria with regard to neurotransmitter metabolism and brain function. *C. albicans* and *K. pneumoniae* were administered (i.g.) to normal C57 mice as monotherapy or in combination. Expression of AChE, AMP, CHRNA1, CHRNB1, and GAD65 was changed in the intestine (*p* < 0.05) (Fig. 9A) and brain (Fig. 9B), especially in the *C. albicans-*treated group in the intestine. These data indicated that changes in two core microorganisms influenced GM-mediated compounds and neurotransmitters.

## Discussion

Increasing numbers of studies have indicated that the GM is closely related to disorders of the nervous system, including autism^31^, depression^32^, schizophrenia^33^, anorexia nervosa^34^, multiple sclerosis^35^, epilepsy^36^, PD^37^, and AD^18^. In recent years, evidence has shown that circRNAs may be important for brain function: CDR1 is found at a high level in mammalian neurons^38^, and the circRNA transcripts from neuron genes can accumulate during aging in *Drosophila*^39^. Hentze and colleagues showed that circRNAs are dynamic during the activities of daily life^40^. Also, circRNAs are expected to be biomarkers or drug targets for neurodegenerative diseases, and studies have shown that the CDR1 level is reduced in AD^38^. Here, we demonstrated that gut dysbacteriosis can implicate the brain circRNA-sequencing directly or indirectly.

Previously, using in an overexpressed *circNF1-419* adeno-associated virus system, we showed that circRNAs in the brain influenced the cholinergic system of the brain, and changed the GM composition, intestinal homeostasis/physiology, and even the GM trajectory in newborn mice^41^. Another system, *circZCCHC11* (mmu_*circ_0001239*), showed a “sponge” miRNA function and mediated a series of chain reactions in the brain, which then influenced gut function and GM engraftment from their parent^41^. Those data demonstrated a link between circRNAs and the GM, enlarged the microbiome–transcriptome linkage library, and provided additional information on the gut–brain axis. Those data and the observations mentioned above indicated that the GM influences the conversion rate of choline to TMAO, and that the TMAO level influences the cholinergic system. In an additional experiment, expression of some circRNAs in the BV2 cell line was measured after incubation with TMAO, which indicated that some circRNAs might be sensitive responsive signaling molecules to TMAO. We concluded that microbial metabolites influenced the formation and degradation of host circRNAs.

Studies have demonstrated that catalyzes the oxidative deamination of biogenic and xenobiotic amines, and has important functions in the metabolism of neuroactive and vasoactive amines in the CNS and peripheral tissues. For example, MAOA preferentially oxidizes biogenic amines such as 5-HT, norepinephrine, and epinephrine. MAOB preferentially degrades benzylamine and phenylethylamine. COMT catalyzes the transfer of a methyl group from S-adenosylmethionine to catechol substrates such as the neurotransmitters dopamine, epinephrine, and norepinephrine, and degrades catecholamine transmitters^42^. All of the above-mentioned monoamine neurotransmitters have indispensable roles in the regulation of CNS functions.

We found that the levels of MAOA, MAOB, and COMT in BV2 cells were affected after TMAO administration, and that the levels of some monoamine neurotransmitters were changed in mice fed the HSHF diet. These data suggested that behavior and stress would be influenced and that, in these processes, the GM had an accelerant role. Therefore, we concluded that the GM decided the transformation efficiency from choline to TMAO. The latter then influenced the levels of MAOA, MAOB, and COMT, which participate in the regulation of metabolism of neuroactive and vasoactive amines in the CNS and peripheral tissues. How circRNAs are involved in this process merits further investigation.

A major goal of any microbiome study is to move beyond correlation, and parse out potential routes of communication/interaction between the host and its resident bacteria. To identify their communication/interaction, the species level was identified using metagenomics sequencing. Twelve species of Archaea were detected, including *Methanobrevibacter* sp. *AbM4* (781.63-fold changes compared with control, Fig. S4A), *Methanosarcina* sp. *MTP4* (1.21-fold, Fig. S4A), and especially *Methanosalsum zhilinae*, *Methanomethylovorans hollandica*, *Candidatus Methanomethylophilus alvus,* and *Thermococcus cleftensis*, which were new residents (Fig. S4A). Most are methanogens (anaerobic prokaryotes from the domain Archaea that utilize hydrogen to reduce carbon dioxide, acetate, and various methyl compounds to methane^43^). Methanogens described in the human microbiota include Euryarchaeota (including *M. smithii*, *M. oralis*, *M. arbophilus*, *M. massiliensis*, *M. luminyensis*, *M. stadtmanae*, *Ca. M. alvus*, and *Ca. M. intestinalis*^44^). Methanogens are emerging pathogens associated with abscesses in the brain and muscles^44^. They have been implicated in dysbiosis of the oral microbiota, periodontitis, and peri-implantitis^43^. They have also been associated with dysbiosis of the digestive-tract microbiota linked to metabolic disorders (anorexia, malnutrition, and obesity) and with lesions of the digestive tract (colon cancer)^43^. One clinical investigation showed that a negative association between methane concentrations in breath and anthropometric biomarkers of obesity^45^, and that methane significantly decreased the neurological deficit induced by cerebral ischemia and reperfusion *via* the antioxidant pathway of PI3K/Akt/HO-1^46^. Special diets have been used to change anaerobic prokaryotes to involve the digestive and nervous systems^47^. ACh is a neurotransmitter in mammalian central and peripheral cholinergic nervous systems. However, it is also widely expressed in non-neuronal animal tissues as well as in plants, fungi, and bacteria, where it is likely involved in the transport of water, electrolytes and nutrients^48^. With the changes in levels of methanogens, based on histopathology, we found that the colon and brain was damaged; neurotransmitter levels were also changed.

Mycobiota are crucial for human health^49^. Surprisingly, a small number of species can trigger huge changes in the human body^50^. Dysbiosis of and invasion by mycobiota can cause disease in different parts of the body^51,52^. Meanwhile, the body also produces corresponding immune changes upon mycobiota infection^53^. Several recent studies have made a connection between intestinal mycobiota and the human immune system^52,53^. In HSHF-diet mice, 10 species of Eukaryota were detected. For example, the relative abundance of *Kluyveromyces lactis*, *Leishmania major*, *Saccharomycetales* species and *Theileria orientalis* was different (*p* < 0.05) (Fig. S4B). *C. albican*s is a very common yeast found in the gastrointestinal tract and oral cavity, and can occasionally cause oral-cavity ulcers. *C. albican*s can pass readily through the blood–brain barrier, where it can cause asymptomatic infection in the cortex, form fungal- induced glial granulomas, and cause short-term memory disorders^54^. *Candida dubliniensis* is an opportunistic fungal pathogen. Bacher and colleagues revealed that human immunity based on T-helper 17 cells against fungi is reliant on cross-reactivity against *C. albicans*^53^. Witchley and coworkers showed that programs of *C. albicans* morphogenesis control the balance between gut commensalism and invasive infection^55^. Neuronal ACh and non-neuronal ACh have been demonstrated to modulate the inflammatory response: ACh protects against *C. albicans* infection by inhibiting biofilm formation and promoting hemocyte function in a model of *Galleria mellonella* infection^56^. Hence, *C. albicans* and *C. dubliniensis* seem to have important roles in host immunity. In comparison with normal groups, the relative abundance of *C. dubliniensis* was increased (Fig. S4B), and levels of proinflammatory mediators were upregulated, upon consumption of the HSHF diet (Fig. 3). Also, in *C. albicans*- and *K. pneumoniae-*treated C57 mice, expression of AChE, AMP, CHRNA1, CHRNB1, and GAD65 were changed in the intestine (Fig. 9A) and brain (Fig. 9A), especially in the *C. albicans-*treated group in the intestine. These data indicated that changes in a few microorganisms could influence microbiota-mediated compounds (including neurotransmitters).

Cooperation within the GM is complex and important for human health, such as mood and bodyweight control. In most cases, the intestinal flora cooperate and influence each other. Cooperative phenotypes are thought to be at the core of microbial-community functions, including through quorum sensing, biofilm formation, and antibiotic resistance^57,58^. We found that with an increase in some *Saccharomyces* species who converted sugars to carbon dioxide and ethanol, the abundance of some methanogens was increased. The reason may have been because the metabolism of alcohol imbalances the proportion of NADH to NAD, galactose tolerance, TG synthesis, and lipid peroxidation (Figs. 3 and 7). Xu and colleagues revealed that chronic exposure to alcohol-induced GM dysbiosis, and was correlated with neuropsychic behaviors^59^. Furthermore, a metabolite from *Saccharomyces*, acetic acid, is the growth substrate of methanogens. The cooperation of these two microorganisms promotes food digestion/absorption, energy storage, and the accumulation of some harmful products^60^. Studies have demonstrated that the PI3K-I/Akt–mTOR signaling network can regulate anabolic processes such as the synthesis of lipids, fatty acids, and nucleotides, and requires an abundant supply of reducing power in the form of NADPH, and the growth factor-stimulated PI3K–Akt–mTORC1 signaling network^61,62^, and the NADPH oxidase nox can be derived by gut microbiome^63^.

Twenty-two species of viruses were detected (Fig. S4C). The numbers of most of them were increased in the HSHF-diet group: *Aureococcus anophagefferens* virus, *Glypta fumiferanae ichnovirus*, *Chrysochromulina ericina* virus, *mouse mammary tumor* virus, *Moloney murine sarcoma* virus, *murine leukemia-related retroviruses*, *murine leukemia* virus, *Mus musculus mobilized endogenous polytropic provirus*, *RD114 retrovirus*, *Shamonda orthobunyavirus*, *Tomato spotted wilt orthotospovirus*, *Cyprinid herpesvirus 1*, *Cyprinid herpesvirus 3*, *Abalone herpesvirus Victoria/AUS/2009*, *Cadicivirus A* and *Lactobacillus prophage Lj771*. Analyses of circRNA sequences showed that the number of circRNAs with downregulated expression was much greater than the number of circRNAs with upregulated expression in HSHF diet-group mice. An identical trend was reported by Liu and colleagues. They found that circRNAs could be degraded by RNase L if induced by polyinosinic:polycytidylic acid or infected by a virus^64^. Furthermore, expression of retinoic acid-inducible gene I supported the notion that too much of a HSHF diet affects immunity and increases the risk of virus invasion (*p* < 0.05) (Fig. S4D). Studies have revealed that *Herpesvirus* species are associated with AD^65^. Therefore, gut dysbacteriosis appears to be a critical factor in inducing changes in the circRNA-expression profile in the brain. Our results also suggest that the stability of circRNAs in terms of structure and quantity is needed for health (i.e., circRNAs may have non-negligible roles in physiological function/regulation in the organism). Also, studies have revealed that the bacteria, diet, and host genes are related to the invasion, infection degree, and drug resistance of viruses^66,67^.

A total of 622 species of bacteria were detected. The relative abundance of species was changed obviously in the model group (Fig. S5). Changes were observed in the abundance of *Bifidobacterium*, *Lactobacillus*, *Bacteroides*, *Prevotella*, *Streptococcus* and *Clostridium* species (Figure S5A–D). With an increase of *Lactobacillus prophage Lj771* abundance (Fig. S4C), the abundance of most of the species of *Lactobacillus* was reduced significantly, including that of *L. acidipiscis*, *L. agilis*, *L. allii*, *L. amylophilus*, *L. amylovorus*, *L. brevis*, *L. coryniformis*, *L. fermentum*, *L. gasseri*, *L. ginsenosidimutans*, *L. helveticus*, *L. jensenii*, *L. johnsonii*, *L. kefiranofaciens*, *L. paraplantarum*, *L. pentosus*, *L. plantarum*, *L. reuteri*, *L. rhamnosus*, *L. Ruminis* and *L. salivarius*, which were found only in the control group (Fig. S4A–D). Simultaneously, the abundance of *Bifidobacterium* species, including that of *B. adolescentis*, *B. asteroids*, *B. bifidum*, *B. breve*, and *B. pseudocatenulatum*, was also inhibited. In contrast, the abundance of most *Bacteroides* species was increased, including *B. caccae*, *B. caecimuris*, *B. cellulosilyticus*, *B. dorei*, *B. fragilis*, *B. helcogenes*, *B. ovatus*, *B. salanitronis*, *B. Thetaiotaomicron* and *B. vulgatus*. Phages may have been the reason why the ratio of *Bifidobacterium* species and *Lactobacillus* species was imbalanced in mice fed the HSHF diet (Fig. 6E). The abundance of *Bifidobacteriaceae* species and *Lactobacillaceae* species is crucial for homeostatic balance in the intestine. The relative abundance of *Lactobacillaceae* species was increased and that of *Bifidobacteriaceae* species was decreased in APP/PS1 mice (Fig. 6E). These data provide evidence that probiotics are beneficial for health under certain physiological conditions.

The abundance of members of the family Lachnospiraceae (Fig. S5), including *Butyrivibrio hungatei*, *Anaerostipes hadrus*, *Butyrivibrio proteoclasticus*, *Blautia* sp. *YL58*, *Herbinix luporum*, *Blautia hansenii*, *Roseburia hominis*, *Lachnoclostridium phocaeense*, [Clostridium] *bolteae*, *phytofermentans*, *Lachnoclostridium Lachnoclostridium* sp. *YL32* and *Pediococcus pentosaceus*, was reduced in the intestinal contents of mice fed the HSHF diet (Fig. S5A–D). These Lachnospiraceae members encode a composite inositol catabolism-butyrate biosynthesis pathway, the presence of which is associated with a lower risk of host metabolic disease^68^. Members of the Lachnospiraceae family are among the main producers of short-chain fatty acids. Different taxa of Lachnospiraceae are also associated with different intra- and extraintestinal diseases^69^. Supplement the butyrate-producing Lachnospiraceae is beneficial for the intestinal barrier^70^, probiotic for treating stress-induced visceral hypersensitivity^71^, the butyrate-producing species *R. inulinivorans* includes strains able to grow on inulin and FOS in pure culture^72^. Furuya and colleagues revealed that *Blautia hansenii* can hydrolyze glucosylceramide to ceramide in plants^73^. Previously, we showed that *circNF1-419* could regulate the synthesis and metabolism of ceramides, and the changed ceramides were enriched in three signaling pathways (neurotrophin, sphingolipid, and adipocytokine)^41^. We could conclude that the couples of *Blautia* and ceramide, butyrate biosynthesis bacteria, and butyrate might be one of interactions between circRNA and microbiome-gut-brain axis, but we still need much more evidence.

*Peptostreptococcaceae*, a family within the order *Clostridiales* (Fig. S5), includes the genera *Peptostreptococcus*, *Acetoanaerobium, Proteocatella, Sporacetigenium*, *Filifactor,* and *Tepidibacter*. The genera *Acetoanaerobium, Sporacetigenium* and *Proteocatella* are monospecific. Representatives of the family have different cell morphology, which varies from cocci to rods and filaments. Species of *Filifactor*, *Proteocatella, Sporacetigenium*, and *Tepidibacter* form endospores. All members of the family are anaerobes with have a fermentative type of metabolism. The genus *Tepidibacter* contains moderately thermophilic species. Members of *Peptostreptococcaceae* are found in different habitats, including the human body, manure, soil, and sediments. Species of *Peptostreptococcus* and *Filifactor* are components of the human oral microbiome^74^. Metagenomics sequencing showed that the relative abundance of *Peptoclostridium acidaminophilum* and *Clostridioides difficile* was increased in HSHF diet-fed mice. *P. acidaminophilum* can ferment amino acids^75^. *C. difficile* is a spore-forming, anaerobic, intestinal pathogen that causes severe diarrhea that can lead to death^76^. *P. acidaminophilum* and *C. difficile* had a negative correlation with expression of *Tpm3* and *Dusp6*, and a positive correlation with expression of 5-HT and 5-HIAA, but the mechanism of action needs further analysis. Bacteria can produce a range of major neurotransmitters, and substantial evidence has accumulated around the microbiota-mediated influence of those compounds^13^. However, the microbiota can also influence levels of neurotransmitters, including histamine, gasotransmitters, neuropeptides, steroids, and endocannabinoids.

## Conclusions

We demonstrated again that consumption of a HSHF diet-induced dysbacteriosis, damaged the intestinal tract, and changed the neurotransmitter metabolism in the intestine and brain. Our new findings were that consumption of a HSHF diet caused changes in brain function and circRNA profiles. Additional experiments found that the GM byproduct of TMAO could degrade some circRNAs, and the basal level of the GM decided the *c*onversion rate of choline to TMAO. A change in the abundance of *C. albicans* and/or *K. pneumoniae* could influence the cholinergic system. These findings demonstrate a new link between metabolism, brain circRNAs, and the GM, enlarge the microbiome–transcriptome linkage library and provide more information on the gut–brain axis.

### Study limitations

There are several strengths with this study, but there are also limitations. For example, the study lacks a rescue test with large number of single bacteria, so we have not fully identified the one-on-one communication/interaction mechanism between neurotransmitter, circRNA and single bacteria, and therefore could not find the signaliccgeted strains in germ-free mice with multi-omics studies to reveal the interaction of neurotransmitter and circRNAs on the microbiome-gut-brain axis, or knock-out mice are needed in the future for positive validations.

### Key resources table

**Table:** 

REAGENT or RESOURCE	SOURCE	IDENTIFIER
Antibodies		
Anti-IBA-1	Proteintech	10904-1-AP
Anti-GFAP	Proteintech	20746-1-AP
Anti-AchE	Proteintech	17975-1-AP
Anti-AMP	Proteintech	13379-1-1P
Anti-CHRNA1	Proteintech	10613-1AP
Anti-CHRNB1	Proteintech	11553-1-AP
Anti-PPAR-γ	Proteintech	11643-1-AP
Anti-TNF-α	Abcam	GR168358-1
Anti-NF-κB p65	Abcam	16502
Anti-IL-2	Proteintech	60306-1-Ig
Anti-MAOA	Proteintech	10539-1-AP
Anti-MAOB	Proteintech	12602-1-AP
Anti-COMT	Proteintech	14754-1-AP
Anti-RIG-I	Affinity	DF6107
β-Actin (13E5)	CST	4970S
GAPDH	Abcam	GR3207992-4

**Table:** 

Chemicals		
Hematoxylin	Servicebio	G1004
Eosin	Servicebio	G1001
Color separation solution	Servicebio	G1039
Diaminobenzidine (DAB)	Servicebio	G1212
Goat anti-rabbit lgG(H + L) HRP	Affinity	S0001
Citrate buffer pH 6.0	Servicebio	G1202
Anhydrous ethanol	Guangzhou Guanghua Sci-Tech co., Ltd	1.17113.023
Penicillin streptomycin solution	CORNING	30002304
Phosphate-buffer saline	CORNING	19117004
Fetal bovine serum	Gibco	1932595
Trimethylamine N-Oxide anhydrous	TOKYO CHEMICAL INDUSTRY CO., LTD	3EVJG-TN
0.25% Trypsin-EDTA (1×)	Gibco	2042337
DMEM basic (1×)	Gibco	8119090

**Table:** 

Critical Commercial Assays		
LDL-C Kit	Nanjing Jiancheng Bioengineering Institute	20180512
HDL-C Kit	Nanjing Jiancheng Bioengineering Institute	20180508
TG Kit	Nanjing Jiancheng Bioengineering Institute	20180609
T-CHO Kit	Nanjing Jiancheng Bioengineering Institute	20190523
ACH Kit	Nanjing Jiancheng Bioengineering Institute	20190505
A-CHE Kit	Nanjing Jiancheng Bioengineering Institute	20190318
RNAiso Plus	TaKaRa	9108
PrimeScript™ RT reagent kit	TaKaRa	RR047A
PrimeScript™ RT reagent kit (RT-PCR)	TaKaRa	RR037A
TB Green™ Premix Ex Taq™ II	TaKaRa	RR820A
Nissol stain kit	Servicebio	G10020
Silver staining kit	Servicebio	G10021

**Table:** 

Deposited Data		
RNA sequencing data	This paper	PRJNA553830
circRNA sequencing data	This paper	PRJNA553830

Software and algorithms		
GraphPad priam	GraphPad software	N/A
Image J	NIH	N/A
SIMCA	Umetrics AB	N/A

**Table:** 

Others		
Choline chloride	Aladdin	C108897
High sugar & fat diet	Beijing HFK Bioscience Co., LTD	(2014)06059
Standard chow	Beijing HFK Bioscience Co., LTD	(2014)06057

## Supplementary information

Supplemental Information

Figure S1

Figure S2

Figure S3A

Figure S3B

Figure S4

Figure S5A-B

Figure S5C-D

## Data Availability

Data collection and sharing for this project was funded by the National Natural Science Foundation of China.

## References

[1] Chen H (2019). A forward chemical genetic screen reveals gut microbiota metabolites that modulate host physiology. Cell.

[2] Rooks MG, Garrett WS (2016). Gut microbiota, metabolites and host immunity. Nat. Rev. Immunol..

[3] Torres-Fuentes C, Schellekens H, Dinan TG, Cryan JF (2017). The microbiota-gut-brain axis in obesity. Lancet Gastroenterol. Hepatol..

[4] Westfall S (2017). Microbiome, probiotics and neurodegenerative diseases: deciphering the gut brain axis. Cell. Mol. Life Sci..

[5] Stilling RM (2016). The neuropharmacology of butyrate: The bread and butter of the microbiota-gut-brain axis?. Neurochem. Int..

[6] Reinshagen M (2019). Neuropods übermitteln Informationen über Nahrungsmittel im Darm über vagale Neuronen in Millisekunden and as Gehirn. Z. f.ür. Gastroenterologie.

[7] Liu P (2019). Altered microbiomes distinguish Alzheimer’s disease from amnestic mild cognitive impairment and health in a Chinese cohort. Brain Behav. Immun..

[8] Li B (2019). Mild cognitive impairment has similar alterations as Alzheimer’s disease in gut microbiota. Alzheimers Dement.

[9] van Kessel SP (2019). Gut bacterial tyrosine decarboxylases restrict levels of levodopa in the treatment of Parkinson’s disease. Nat. Commun..

[10] O’Neill C (2019). Gut microbes metabolize Parkinson’s disease drug. Science.

[11] Fujisaka S (2018). Diet, genetics, and the gut microbiome drive dynamic changes in plasma metabolites. Cell Rep..

[12] Thaiss CA (2018). Hyperglycemia drives intestinal barrier dysfunction and risk for enteric infection. Science.

[13] Strandwitz P (2018). Neurotransmitter modulation by the gut microbiota. Brain Res.

[14] Olson CA (2018). The gut microbiota mediates the anti-seizure effects of the ketogenic diet. Cell.

[15] Strandwitz P (2019). GABA-modulating bacteria of the human gut microbiota. Nat. Microbiol..

[16] Worthy SE (2018). Identification of attractive odorants released by preferred bacterial food found in the natural habitats of C. elegans. PLoS ONE.

[17] O Donnell MP, Fox BW, Chao P, Schroeder FC, Sengupta P (2020). A neurotransmitter produced by gut bacteria modulates host sensory behaviour. Nature.

[18] Jiang C, Li G, Huang P, Liu Z, Zhao B (2017). The gut microbiota and Alzheimer’s disease. J. Alzheimers Dis..

[19] Kaelberer MM (2018). A gut-brain neural circuit for nutrient sensory transduction. Science.

[20] Zheng P (2019). The gut microbiome from patients with schizophrenia modulates the glutamate-glutamine-GABA cycle and schizophrenia-relevant behaviors in mice. Sci. Adv..

[21] Liu R (2017). Gut microbiome and serum metabolome alterations in obesity and after weight-loss intervention. Nat. Med..

[22] Saffouri GB (2019). Small intestinal microbial dysbiosis underlies symptoms associated with functional gastrointestinal disorders. Nat. Commun..

[23] Jeong JH, Lee DK, Jo Y (2017). Cholinergic neurons in the dorsomedial hypothalamus regulate food intake. Mol. Metab..

[24] Hampel H (2018). The cholinergic system in the pathophysiology and treatment of Alzheimer’s disease. Brain.

[25] Kwon YH (2019). Modulation of gut microbiota composition by serotonin signaling influences intestinal immune response and susceptibility to colitis. Cell. Mol. Gastroenterol. Hepatol..

[26] Cox MA (2019). Choline acetyltransferase–expressing T cells are required to control chronic viral infection. Science.

[27] Zhu W (2016). Gut microbial metabolite TMAO enhances platelet hyperreactivity and thrombosis risk. Cell.

[28] Vogt NM (2018). The gut microbiota-derived metabolite trimethylamine N-oxide is elevated in Alzheimer’s disease. Alzheimers Res. Ther..

[29] Pecoraro V (2017). A subnanomolar concentration of Pituitary Adenylate Cyclase-Activating Polypeptide (PACAP) pre-synaptically modulates glutamatergic transmission in the rat hippocampus acting through acetylcholine. Neuroscience.

[30] Subramaniam S, Fletcher C (2018). Trimethylamine N-oxide: breathe new life. Br. J. Pharm..

[31] Doenyas C (2018). Gut microbiota, inflammation, and probiotics on neural development in autism spectrum disorder. Neuroscience.

[32] Lach G, Schellekens H, Dinan TG, Cryan JF (2018). Anxiety, depression, and the microbiome: a role for gut peptides. Neurotherapeutics.

[33] Xu R (2020). Altered gut microbiota and mucosal immunity in patients with schizophrenia. Brain, Behav., Immun..

[34] Seitz J (2019). The impact of starvation on the microbiome and gut-brain interaction in anorexia nervosa. Front. Endocrinol..

[35] Camara-Lemarroy CR, Metz L, Meddings JB, Sharkey KA, Wee YV (2018). The intestinal barrier in multiple sclerosis: implications for pathophysiology and therapeutics. Brain.

[36] Dahlin M, Prast-Nielsen S (2019). The gut microbiome and epilepsy. EBioMedicine.

[37] Sampson TR (2016). Gut microbiota regulate motor deficits and neuroinflammation in a model of Parkinson’s disease. Cell.

[38] Memczak S (2013). Circular RNAs are a large class of animal RNAs with regulatory potency. Nature.

[39] Weigelt CM (2020). An insulin-sensitive circular RNA that regulates lifespan in drosophila. Mol. Cell..

[40] Hentze MW, Preiss T (2013). Circular RNAs: splicing’s enigma variations. EMBO J..

[41] Diling C (2020). CircNF1-419 improves the gut microbiome structure and function in AD-like mice. Aging (Albany NY).

[42] Tong J (2017). Brain monoamine oxidase B and A in human parkinsonian dopamine deficiency disorders. Brain.

[43] Sogodogo E, Drancourt M, Grine G (2019). Methanogens as emerging pathogens in anaerobic abscesses. Eur. J. Clin. Microbiol Infect. Dis..

[44] Drancourt M (2017). Evidence of archaeal methanogens in brain abscess. Clin. Infect. Dis..

[45] Wilder-Smith CH, Olesen SS, Materna A, Drewes AM (2018). Breath methane concentrations and markers of obesity in patients with functional gastrointestinal disorders. U. Eur. Gastroenterol. J..

[46] Zhang B, Gao M, Shen J, He D (2017). Inhaled methane protects rats against neurological dysfunction induced by cerebral ischemia and reperfusion injury: PI3K/Akt/HO-1 pathway involved. Arch. Med. Res..

[47] Pimentel M, Lembo A (2020). Microbiome and its role in irritable bowel syndrome. Dig. Dis. Sci..

[48] Yamada T (2005). Expression of acetylcholine (ACh) and ACh-synthesizing activity in Archaea. Life Sci..

[49] Zhang D (2020). The mycobiota of the human body: a spark can start a prairie fire. Gut Microbes.

[50] Schei K (2017). Early gut mycobiota and mother-offspring transfer. Microbiome.

[51] Li XV, Leonardi I, Iliev ID (2019). Gut mycobiota in immunity and inflammatory disease. Immunity.

[52] Richard ML, Sokol H (2019). The gut mycobiota: insights into analysis, environmental interactions and role in gastrointestinal diseases. Nat. Rev. Gastroenterol. Hepatol..

[53] Bacher P (2019). Human anti-fungal Th17 immunity and pathology rely on cross-reactivity against Candida albicans. Cell.

[54] Valentine M, Benade E, Mouton M, Khan W, Botha A (2019). Binary interactions between the yeast Candida albicans and two gut-associated Bacteroides species. Micro. Pathog..

[55] Witchley JN (2019). Candida albicans morphogenesis programs control the balance between gut commensalism and invasive infection. Cell Host Microbe.

[56] Nile C (2019). Repurposing pilocarpine hydrochloride for treatment of Candida albicans infections. mSphere.

[57] Haase S, Haghikia A, Wilck N, Muller DN, Linker RA (2018). Impacts of microbiome metabolites on immune regulation and autoimmunity. Immunology.

[58] Sherwin E, Bordenstein SR, Quinn JL, Dinan TG, Cryan JF (2019). Microbiota and the social brain. Science.

[59] Xu Z (2019). Chronic alcohol exposure induced gut microbiota dysbiosis and its correlations with neuropsychic behaviors and brain BDNF/Gabra1 changes in mice. Biofactors.

[60] Ribas D (2019). The acetate uptake transporter family motif “NPAPLGL(M/S)” is essential for substrate uptake. Fungal Genet. Biol..

[61] Wolfson RL (2016). Sestrin2 is a leucine sensor for the mTORC1 pathway. Science.

[62] Yang H (2017). Mechanisms of mTORC1 activation by RHEB and inhibition by PRAS40. Nature.

[63] Iatsenko I, Boquete JP, Lemaitre B (2018). Microbiota-derived lactate activates production of reactive oxygen species by the intestinal NADPH oxidase nox and shortens Drosophila lifespan. Immunity.

[64] Liu CX (2019). Structure and degradation of circular RNAs regulate PKR activation in innate immunity. Cell.

[65] Eimer WA (2018). Alzheimer’s disease-associated beta-amyloid is rapidly seeded by herpesviridae to protect against brain infection. Neuron.

[66] Dhar D, Mohanty A (2020). Gut microbiota and Covid-19- possible link and implications. Virus Res.

[67] Shkoporov AN (2019). The human gut virome is highly diverse, stable, and individual specific. Cell Host Microbe.

[68] Zeng X (2019). Higher risk of stroke is correlated with increased opportunistic pathogen load and reduced levels of butyrate-producing bacteria in the gut. Front Cell Infect. Microbiol.

[69] Vacca M (2020). The controversial role of human gut Lachnospiraceae. Microorganisms.

[70] Sasaki K (2019). Construction of a model culture system of human colonic microbiota to detect decreasedlachnospiraceae abundance and butyrogenesis in the feces of ulcerative colitis patients. Biotechnol. J..

[71] Zhang J (2019). Beneficial effect of butyrate-producing Lachnospiraceae on stress-induced visceral hypersensitivity in rats. J. Gastroenterol. Hepatol..

[72] Louis P, Young P, Holtrop G, Flint HJ (2010). Diversity of human colonic butyrate-producing bacteria revealed by analysis of the butyryl-CoA:acetate CoA-transferase gene. Environ. Microbiol.

[73] Furuya H, Ide Y, Hamamoto M, Asanuma N, Hino T (2010). Isolation of a novel bacterium, Blautia glucerasei sp. nov., hydrolyzing plant glucosylceramide to ceramide. Arch. Microbiol..

[74] Galperin MY, Brover V, Tolstoy I, Yutin N (2016). Phylogenomic analysis of the family Peptostreptococcaceae (Clostridium cluster XI) and proposal for reclassification of Clostridium litorale (Fendrich et al. 1991) and Eubacterium acidaminophilum (Zindel et al. 1989) as Peptoclostridium litorale gen. nov. comb. nov. and Peptoclostridium acidaminophilum comb. nov. Int. J. Syst. Evol. Micr.

[75] Mei R, Nobu MK, Liu WT (2020). Identifying anaerobic amino acids degraders through the comparison of short‐term and long‐term enrichments. Env. Microbiol. Rep..

[76] Sandhu BK, McBride SM (2018). Clostridioides difficile. Trends Microbiol.

