# Supplementary material for: A diet high in sugar and fat influences neurotransmitter metabolism and then affects brain function by altering the gut microbiota
Source: Transl Psychiatry. 2021 May 27;11:328. doi: 10.1038/s41398-021-01443-2 (PMC8160265; doi:10.1038/s41398-021-01443-2)
Supplement: Supplementary file 1 — Supplemental Information [file 41398_2021_1443_MOESM1_ESM.docx]

# Supplemental Information

## Supplemental Figures


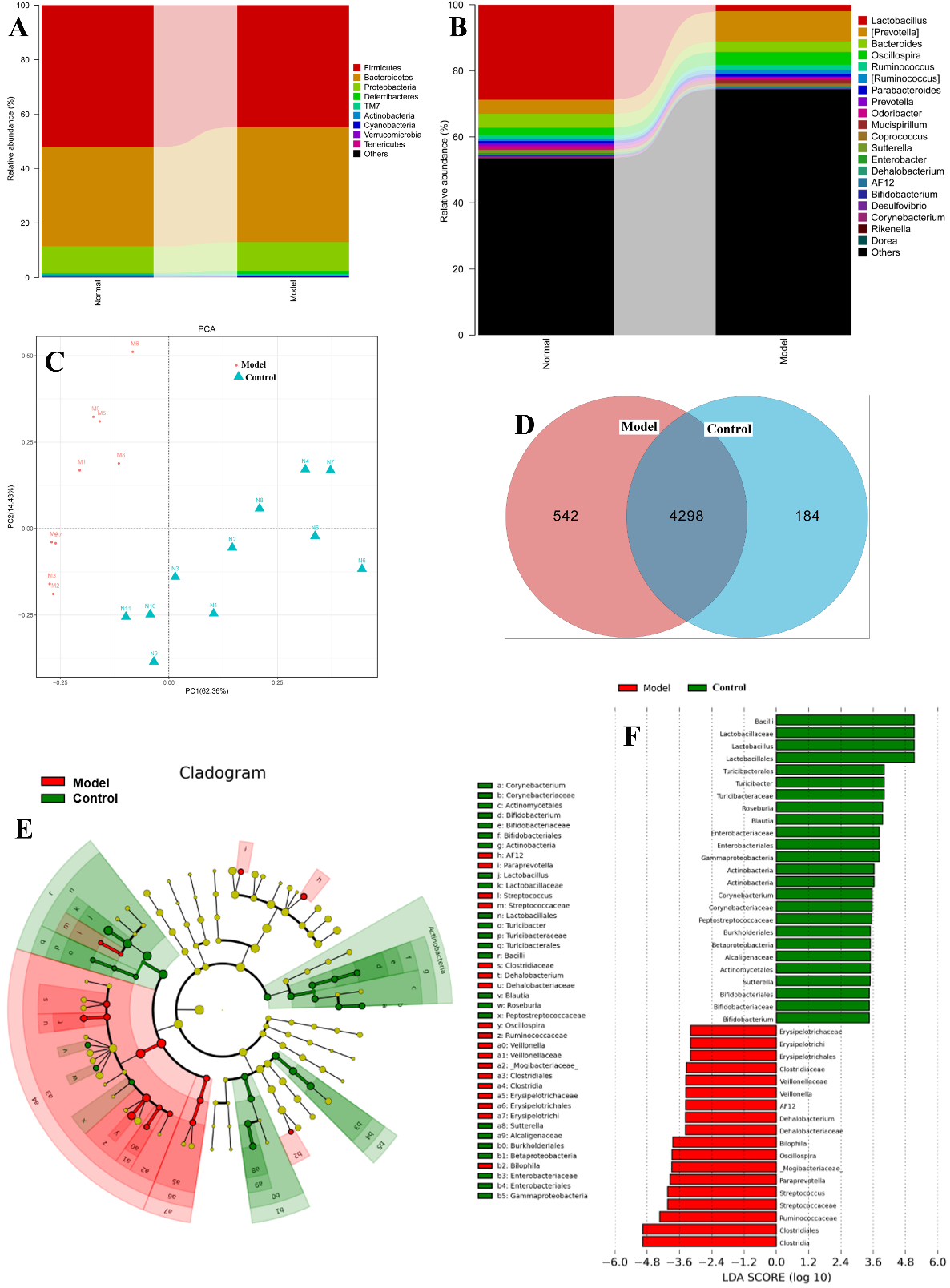


**Figure S1 Gut microbiota analysis on high sugar & fat diet fed mice using 16S rDNA.** (A) the relative abundance of gut microbiota at phylum level; (B) the relative abundance of gut microbiota at genus level; (C) PCA analysis could [distinguish](javascript:;) these two groups of control and high sugar & fat diet fed; (D) Venn diagram showed the common differences between these two groups; (E-F) The dominant species classification tree of OTU using LEfSe analysis. (Linear discriminant analysis, *LDA*>2, *p* < 0.05). Data are presented as the means ± SD of more than 8 independent experiments. **p* <0.05 and ***p* <0.01 *vs*. the model group by one-way ANOVA, followed by the Holm-Sidak test


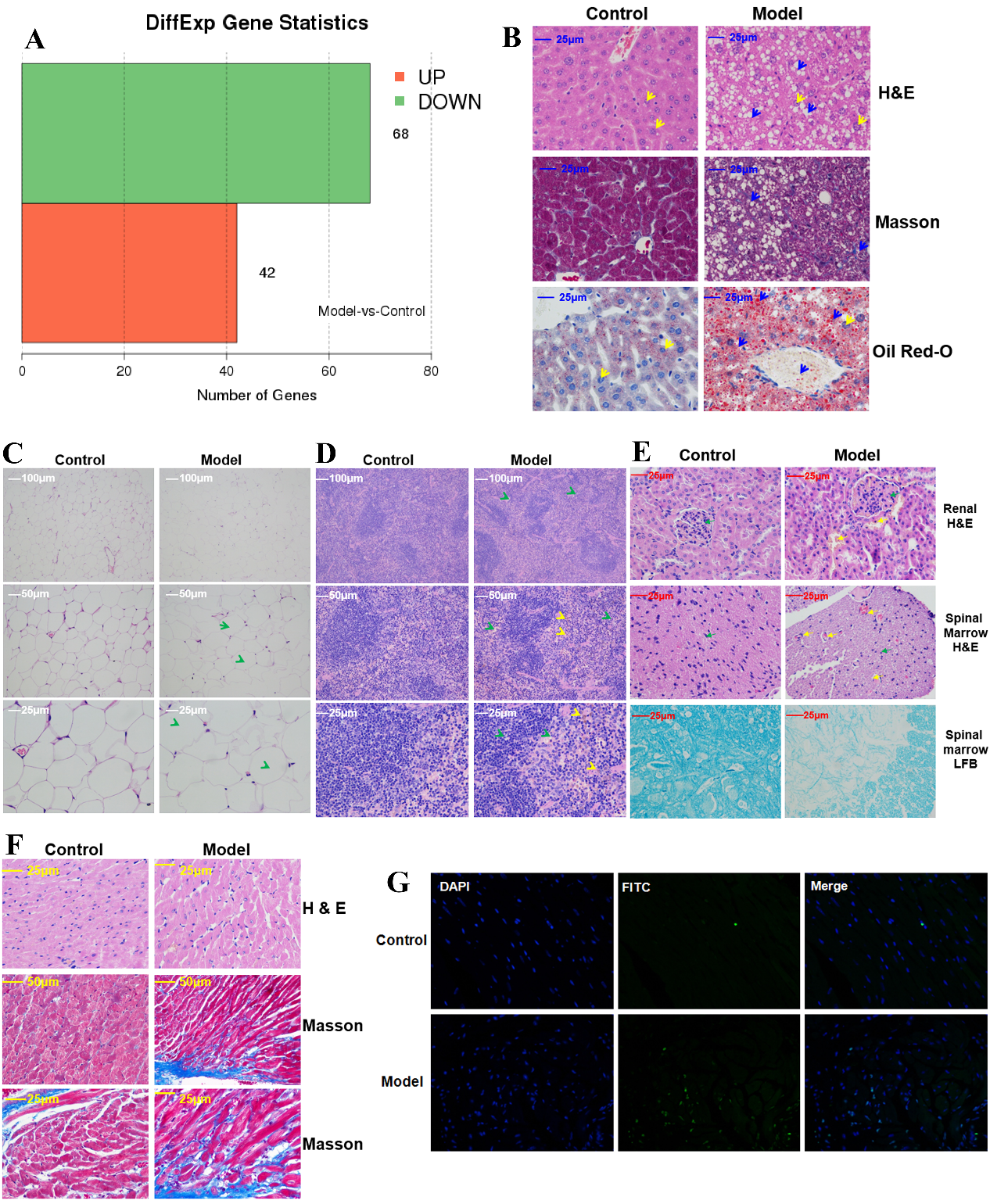


**Figure S2** **The high sugar & fat diet induced dysbacteriosis, then activated inflammation and damaged multiple organs in mice.** A is the RNA-seq of small intestinal tissues in high sugar & fat diet fed mice; B is the livers stained with hematoxylin and eosin (H&E), Masson and oil red O staining (ORO), which the cell shrinkage, cell size reduction and turn rounded, cytoplasmic vacuolar changes, fibrosis, adipose cells increased significantly, which indicated that fatty liver are coupled with dysbacteriosis; C is the pathologic changes on abdominal subcutaneous adipose tissue; and spleen (D), renal (Figure E), spinal marrow (E, H&E, Luxol Fast Blue, LFB), and heart tissues (F, H&E and Masson), which showed that cells of dysbacteriosis mice were shrinkaged, cell size reduced, cytoplasmic vacuolar changed and number in decline, also the apoptosis rate of heart cells (G). Data are presented as the means ± SD of more than 8 independent experiments, and the histopathology data were more than 3. **p* <0.05 and ***p* <0.01 *vs*. the model group by one-way ANOVA, followed by the Holm-Sidak test

**
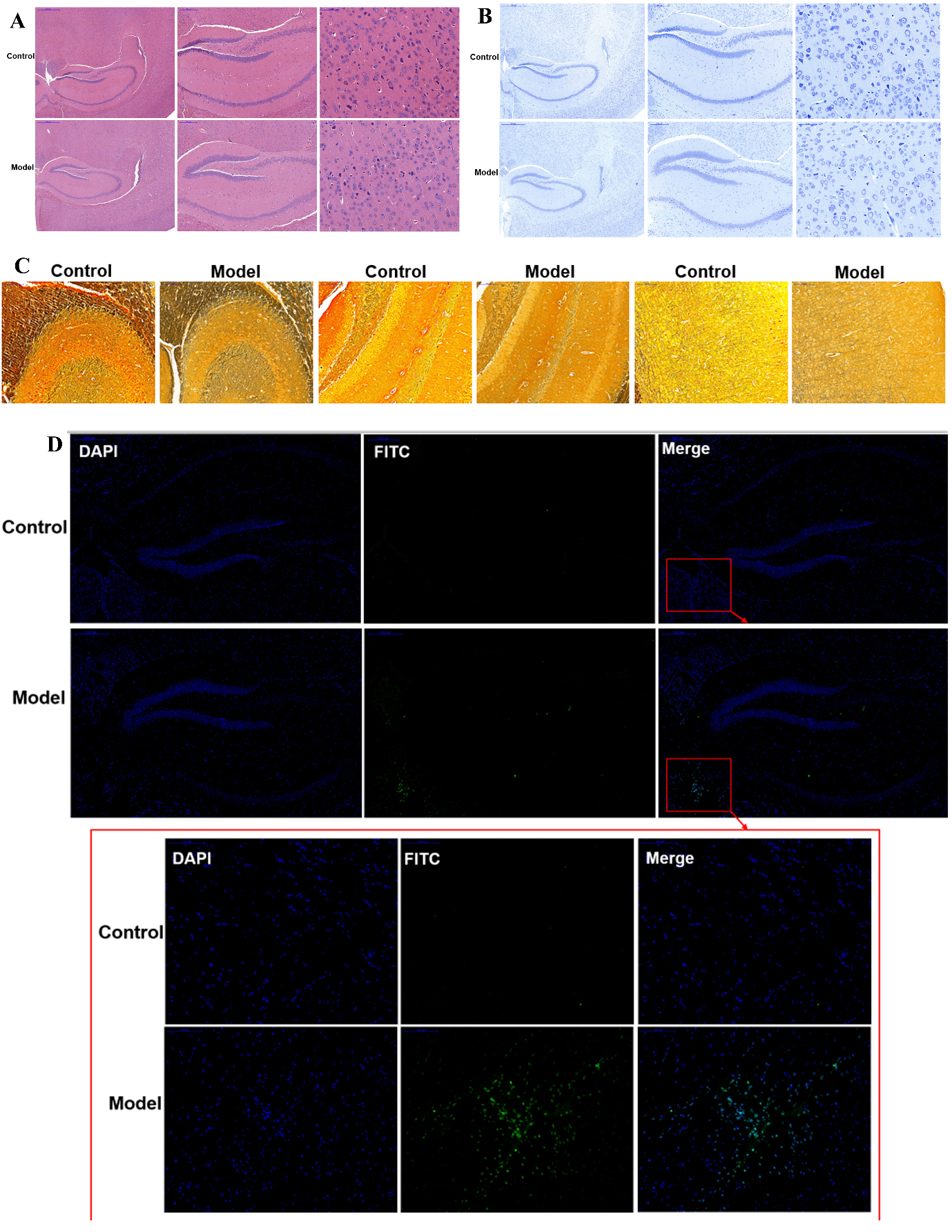
**

**Figure S3A** **Dysbacteriosis implicated brain histopathology in high sugar & fat diet fed mice lasting four months.** (A) Samples were stained using hematoxylin and eosin (H&E); (B) Samples were stained using Nissl staining; (C) Samples were stained using Silver staining;(D) Samples were stained using TUNEL staining. Data are presented as the means ± SD of more than 8 independent experiments, and the histopathology data were more than 3. **p* <0.05 and ***p* <0.01 *vs*. the model group by one-way ANOVA, followed by the Holm-Sidak test

**
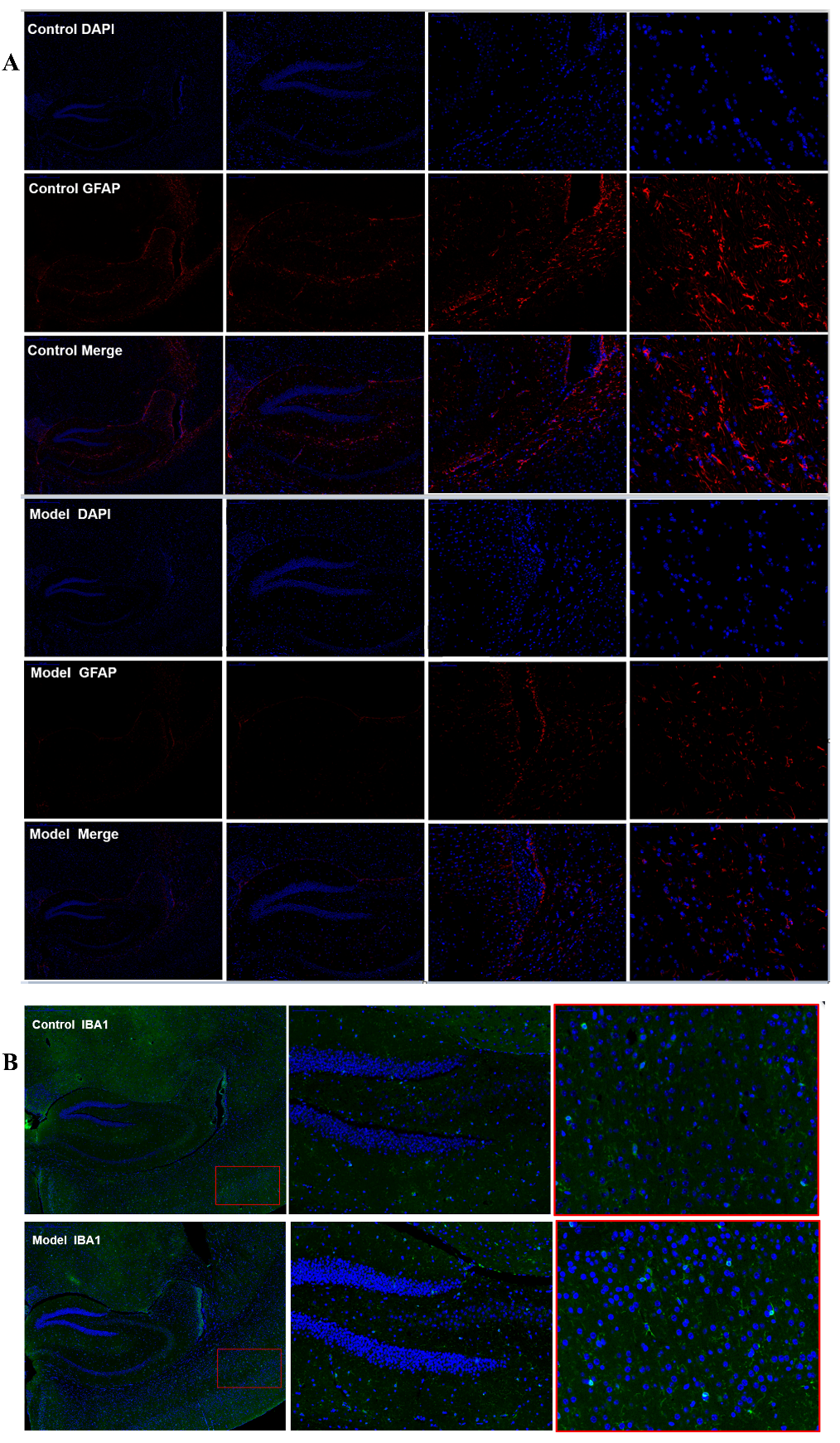
**

**Figure S3B Dysbacteriosis implicated the brain functions and circRNAs sequencing in high sugar & fat diet fed mice lasting four months.** (A) Samples were stained using immunofluorescent antibody of GFAP; (B) Samples were stained using immunofluorescent microglial of IBA-1. Data are presented as the means ± SD of more than 8 independent experiments, and the histopathology data were more than 3. **p* <0.05 and ***p* <0.01 *vs*. the model group by one-way ANOVA, followed by the Holm-Sidak test

**
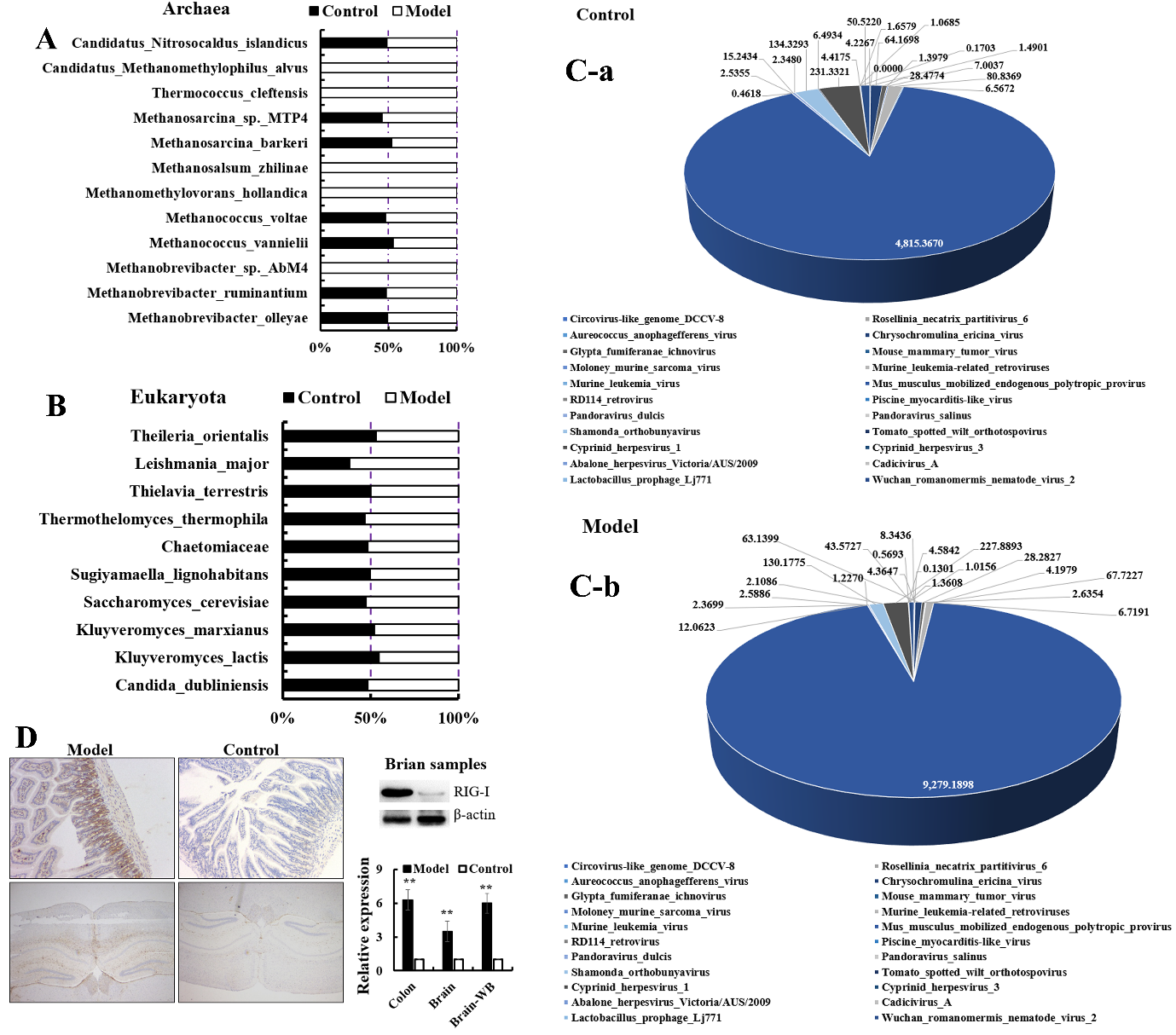
**

**Figure S4** Metagenomic analysis of the mechanism of microorganism species on the gut-brain axis, see also in Figure S5. (A) The relative abundance of Archea *in fimo* of high sugar & fat diet fed mice; (B) The relative abundance of Eukaryota *in fimo* of high sugar & fat diet fed mice; (C) The abundance of virus *in fimo* of high sugar & fat diet fed mice; (D) The relative expression of RIG-I in in the high sugar & fat diet fed mice brain. Data are presented as the means ± SD of 3 independent experiments. ^*^*p* <0.05 and ^**^*p* < 0.01 *vs*. the model group by one-way ANOVA, followed by the Holm-Sidak test

**
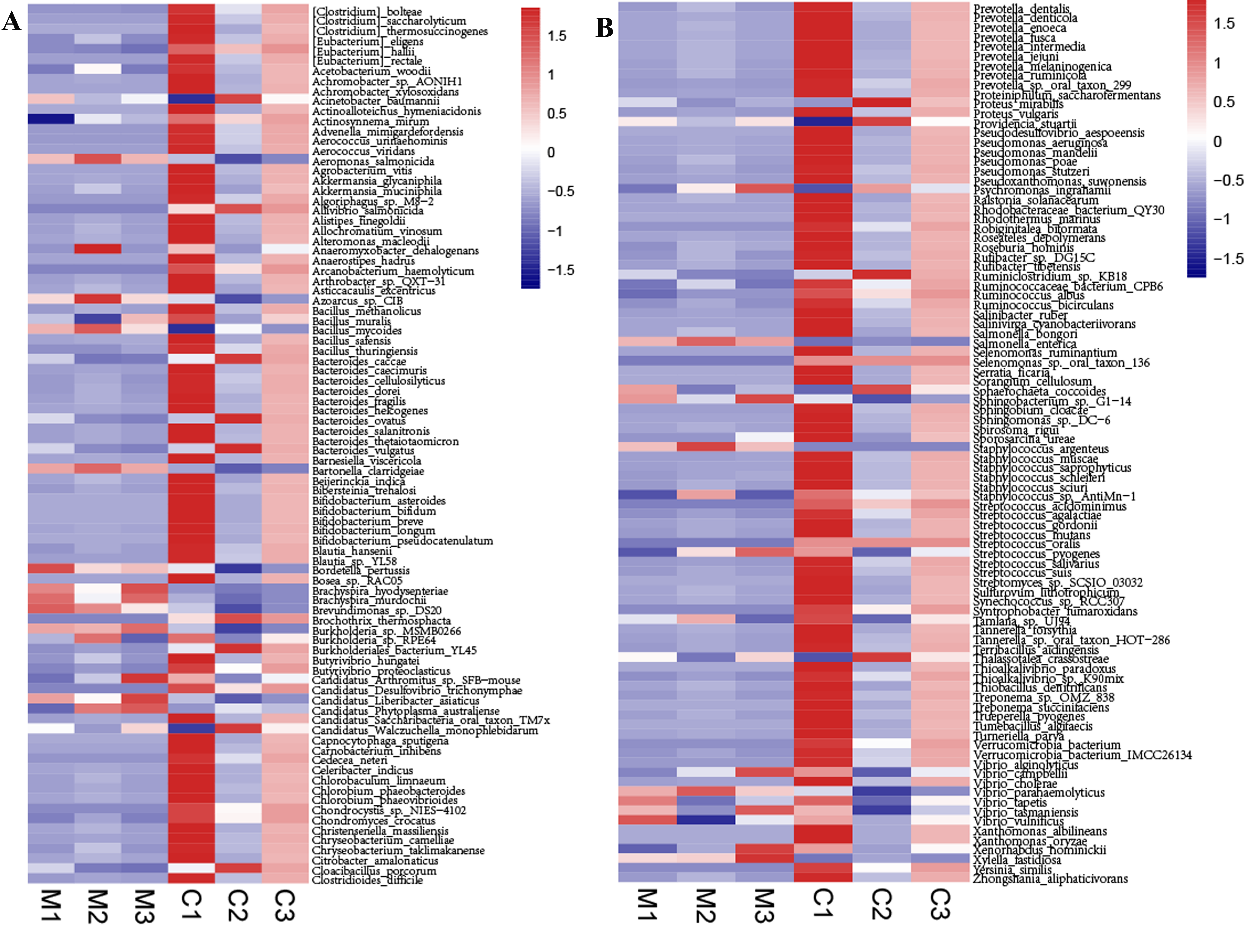
**

**
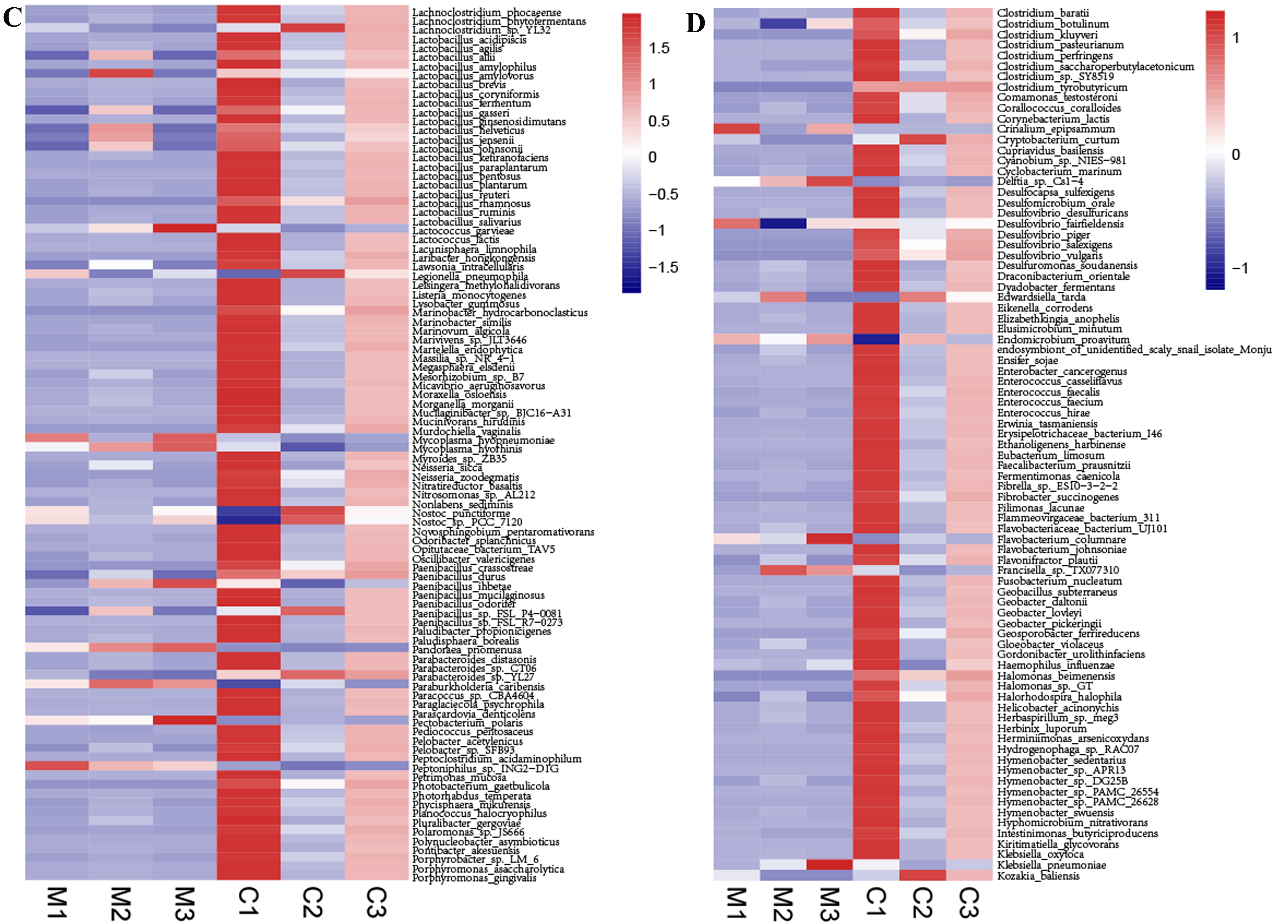
**

**Figure S5** Metagenomic analysis of the bacteria mechanism of action of microorganism species on the gut-brain axis, and there were 622 species of Bacteria detected, and the relative abundance and species were obviously changed in the model group (n=3).

## Supplemental tables

**Table S1** Different expression of mRNAs in the colon of high sugar & fat diet fed induced dysbacteriosis mice (control vs model group, Foldchange > 1.50, *p* < 0.05)

### Table S1

**Table S1 Different expression of mRNAs in the colon of high sugar & fat diet fed induced dysbacteriosis mice (control vs model group, Foldchange > 1.50, *p* < 0.05)**

| **ID** | **Model_count** | **Control_count** | **Model_fpkm** | **Control_fpkm** | **log2(FC)** | **Pvalue** | **FDR** | **significant** | **Symbol** |
| --- | --- | --- | --- | --- | --- | --- | --- | --- | --- |
| ENSMUSG00000102049 | 0.0000 | 33.3333 | 0.0010 | 0.3900 | 7.94 | 2.25E-14 | 5.69E-11 | up | Zbed6 |
| ENSMUSG00000053830 | 0.0000 | 27.7200 | 0.0010 | 0.7100 | 7.78 | 1.34E-05 | 0.003786 | up | - |
| ENSMUSG00000049604 | 0.0000 | 9.6667 | 0.0010 | 0.3100 | 6.29 | 0.00028 | 0.045544 | up | Hoxb13 |
| ENSMUSG00000017723 | 1.0000 | 52.3333 | 0.1133 | 5.9067 | 5.22 | 0.00013 | 0.027373 | up | Wfdc2 |
| ENSMUSG00000091971 | 112.5600 | 4094.0700 | 2.5967 | 85.1200 | 4.93 | 2.07E-24 | 3.67E-20 | up | Hspa1a |
| ENSMUSG00000090877 | 105.5233 | 3694.2167 | 2.5600 | 82.5600 | 4.87 | 1.95E-15 | 5.77E-12 | up | Hspa1b |
| XLOC_010091 | 4.6667 | 98.3333 | 0.0733 | 1.3300 | 4.13 | 5.50E-14 | 1.22E-10 | up | TCONS_00053457 |
| ENSMUSG00000053706 | 1.3867 | 23.8267 | 0.0267 | 0.3900 | 3.86 | 2.25E-06 | 0.000928 | up | B430305J03Rik |
| XLOC_017402 | 12.3333 | 204.0000 | 14.7600 | 248.5100 | 3.82 | 1.09E-17 | 4.81E-14 | up | TCONS_00102194 |
| ENSMUSG00000066867 | 2.6667 | 29.3333 | 0.1367 | 1.2600 | 3.24 | 5.61E-05 | 0.013613 | up | Oas1e |
| ENSMUSG00000044359 | 5.6667 | 45.6667 | 0.0867 | 0.6200 | 2.82 | 6.06E-06 | 0.00211 | up | P2ry4 |
| ENSMUSG00000046697 | 658.6667 | 4973.0000 | 40.0333 | 273.0033 | 2.74 | 3.80E-08 | 2.69E-05 | up | Enpp7 |
| ENSMUSG00000074968 | 5.6667 | 35.0000 | 0.0567 | 0.3033 | 2.42 | 8.57E-05 | 0.018749 | up | Ano3 |
| ENSMUSG00000029657 | 573.3333 | 3506.3333 | 15.1067 | 87.1267 | 2.41 | 9.01E-16 | 3.19E-12 | up | Hsph1 |
| ENSMUSG00000038473 | 40.0000 | 222.1367 | 3.0133 | 15.3133 | 2.31 | 1.06E-05 | 0.003247 | up | Nos1ap |
| ENSMUSG00000041644 | 1581.6667 | 8163.6667 | 31.3833 | 143.6400 | 2.19 | 4.53E-07 | 0.000251 | up | Slc5a12 |
| ENSMUSG00000020229 | 352.6667 | 1823.6667 | 12.6200 | 58.1500 | 2.13 | 8.02E-10 | 8.36E-07 | up | Slc5a4a |
| XLOC_000473 | 38.6667 | 190.6667 | 1.7033 | 7.7133 | 2.07 | 5.71E-05 | 0.013665 | up | TCONS_00003927 |
| ENSMUSG00000003545 | 35.3333 | 161.0000 | 0.7967 | 3.5200 | 1.98 | 8.94E-08 | 5.46E-05 | up | Fosb |
| ENSMUSG00000020682 | 20.0633 | 92.0300 | 0.4767 | 1.7633 | 1.94 | 8.28E-05 | 0.018345 | up | Mmp28 |
| ENSMUSG00000037005 | 844.0000 | 3634.6800 | 17.7767 | 66.4233 | 1.87 | 7.06E-06 | 0.002358 | up | Xpnpep2 |
| ENSMUSG00000024421 | 291.0000 | 1197.3333 | 3.0633 | 11.4667 | 1.86 | 1.21E-05 | 0.003558 | up | Lama3 |
| ENSMUSG00000026726 | 2478.0000 | 10122.6667 | 13.9833 | 50.2700 | 1.81 | 5.23E-09 | 4.63E-06 | up | Cubn |
| XLOC_004873 | 42.3333 | 164.6667 | 8.3067 | 28.7467 | 1.76 | 7.59E-05 | 0.017032 | up | TCONS_00030165 |
| ENSMUSG00000028737 | 713.1667 | 2711.4733 | 14.5300 | 48.4100 | 1.70 | 8.70E-07 | 0.00044 | up | Aldh4a1 |
| ENSMUSG00000021573 | 197.3333 | 659.3333 | 2.9333 | 7.7933 | 1.58 | 1.88E-05 | 0.004931 | up | Tppp |
| ENSMUSG00000042510 | 585.3333 | 2001.6667 | 17.9733 | 53.0667 | 1.57 | 2.30E-08 | 1.85E-05 | up | AA986860 |
| ENSMUSG00000022180 | 2777.6667 | 9312.6667 | 46.7533 | 137.4567 | 1.54 | 6.52E-07 | 0.00034 | up | Slc7a8 |
| ENSMUSG00000034248 | 1629.9633 | 5125.4100 | 21.9900 | 56.2000 | 1.43 | 1.96E-06 | 0.000847 | up | Slc25a37 |
| ENSMUSG00000020893 | 361.3333 | 1100.0000 | 9.8067 | 30.9333 | 1.40 | 0.000186 | 0.033326 | up | Per1 |
| ENSMUSG00000032265 | 1852.6667 | 5498.0000 | 22.0867 | 57.1433 | 1.38 | 9.89E-06 | 0.003128 | up | Fam46a |
| ENSMUSG00000020447 | 8629.0000 | 24776.0000 | 130.5667 | 328.1433 | 1.32 | 3.30E-05 | 0.008244 | up | Npc1l1 |
| ENSMUSG00000001095 | 2533.0000 | 7321.0000 | 75.1800 | 194.9700 | 1.31 | 0.000171 | 0.032548 | up | Slc13a2 |
| ENSMUSG00000074195 | 14008.7567 | 39716.3133 | 328.7233 | 816.7700 | 1.30 | 1.78E-05 | 0.004769 | up | Clca4b |
| ENSMUSG00000025557 | 4650.3333 | 12899.6667 | 103.5433 | 254.2500 | 1.26 | 1.31E-05 | 0.003786 | up | Slc15a1 |
| ENSMUSG00000034320 | 1838.6933 | 5039.6667 | 15.3500 | 36.8833 | 1.25 | 0.000255 | 0.043068 | up | Slc26a2 |
| ENSMUSG00000021250 | 1254.0000 | 3448.6667 | 45.4767 | 111.2400 | 1.23 | 0.000132 | 0.027478 | up | Fos |
| ENSMUSG00000020681 | 32553.3333 | 83294.3333 | 499.7600 | 1098.5467 | 1.16 | 0.000207 | 0.03601 | up | Ace |
| ENSMUSG00000020097 | 6076.6667 | 15008.6667 | 107.1333 | 225.5467 | 1.11 | 0.000263 | 0.043464 | up | Sgpl1 |
| ENSMUSG00000054517 | 238.3333 | 586.6667 | 4.5900 | 9.9967 | 1.10 | 0.000184 | 0.033326 | up | Trim65 |
| ENSMUSG00000054499 | 578.6667 | 1368.0000 | 16.2267 | 33.6700 | 1.05 | 0.000164 | 0.031633 | up | Dedd2 |
| ENSMUSG00000019850 | 339.0000 | 809.0000 | 5.2100 | 11.8600 | 1.04 | 0.000193 | 0.033905 | up | Tnfaip3 |
| ENSMUSG00000000876 | 1019.0000 | 564.3567 | 34.3133 | 16.6867 | -1.05 | 0.000227 | 0.03901 | down | Pxmp4 |
| ENSMUSG00000028307 | 136563.3333 | 73734.6667 | 5138.7467 | 2433.1467 | -1.10 | 0.000258 | 0.043136 | down | Aldob |
| ENSMUSG00000028494 | 3756.0000 | 2004.3333 | 163.3700 | 77.0733 | -1.13 | 0.000137 | 0.027737 | down | Plin2 |
| ENSMUSG00000056737 | 345.3333 | 177.0000 | 23.2200 | 10.9567 | -1.19 | 0.00016 | 0.03121 | down | Capg |
| ENSMUSG00000038370 | 288.0000 | 139.3333 | 14.4400 | 6.2733 | -1.26 | 0.000138 | 0.027737 | down | Pcp4l1 |
| ENSMUSG00000094724 | 888.7933 | 421.0767 | 83.2133 | 34.0900 | -1.32 | 1.89E-05 | 0.004931 | down | Rnaset2b |
| ENSMUSG00000026639 | 1074.3333 | 456.6667 | 20.3600 | 7.5967 | -1.43 | 1.05E-05 | 0.003247 | down | Lamb3 |
| ENSMUSG00000034731 | 79.3333 | 33.6667 | 1.3667 | 0.5033 | -1.44 | 0.000297 | 0.047869 | down | Dgkh |
| ENSMUSG00000021226 | 378.2067 | 156.9833 | 12.8133 | 4.6567 | -1.47 | 6.75E-06 | 0.002301 | down | Acot2 |
| ENSMUSG00000020538 | 6791.6667 | 2679.6667 | 144.7033 | 52.0200 | -1.53 | 1.29E-06 | 0.0006 | down | Srebf1 |
| XLOC_008415 | 870.3333 | 355.3333 | 516.0067 | 217.8200 | -1.54 | 2.27E-05 | 0.005841 | down | TCONS_00045808 |
| ENSMUSG00000028583 | 59.6667 | 23.3333 | 2.8367 | 0.8667 | -1.56 | 0.000175 | 0.032555 | down | Pdpn |
| ENSMUSG00000032418 | 6322.3333 | 2330.3333 | 153.8467 | 52.7267 | -1.65 | 3.99E-06 | 0.001503 | down | Me1 |
| ENSMUSG00000051727 | 321.3333 | 120.6667 | 10.4633 | 3.4567 | -1.67 | 1.09E-05 | 0.003263 | down | Kctd14 |
| ENSMUSG00000090175 | 5504.0233 | 2027.3867 | 183.2700 | 60.1433 | -1.68 | 1.89E-06 | 0.000836 | down | Ugt1a9 |
| ENSMUSG00000002992 | 21952.2567 | 8115.0567 | 4760.8433 | 1662.1300 | -1.68 | 7.97E-06 | 0.002615 | down | Apoc2 |
| ENSMUSG00000045410 | 336.3333 | 121.0000 | 14.6933 | 4.4867 | -1.69 | 9.83E-07 | 0.000484 | down | Akr1e1 |
| ENSMUSG00000054422 | 47933.3333 | 16737.0000 | 14284.5167 | 4917.8400 | -1.75 | 1.35E-05 | 0.003786 | down | Fabp1 |
| ENSMUSG00000010830 | 676.0000 | 229.3333 | 39.3467 | 11.8667 | -1.77 | 2.92E-06 | 0.001176 | down | Kdelr3 |
| ENSMUSG00000029762 | 184.6667 | 60.3333 | 11.3067 | 3.3200 | -1.82 | 6.07E-06 | 0.00211 | down | Akr1b8 |
| ENSMUSG00000010651 | 2768.4900 | 823.8267 | 129.6400 | 35.6433 | -1.95 | 6.08E-07 | 0.000327 | down | Acaa1b |
| ENSMUSG00000005237 | 722.6667 | 220.6667 | 3.4600 | 1.0467 | -1.96 | 1.05E-06 | 0.000503 | down | Dnah2 |
| ENSMUSG00000028655 | 2640.0000 | 756.6667 | 123.0733 | 31.6900 | -1.98 | 7.16E-10 | 7.93E-07 | down | Mfsd2a |
| ENSMUSG00000078650 | 2082.0000 | 607.6667 | 62.2000 | 16.0133 | -2.01 | 4.64E-10 | 5.48E-07 | down | G6pc |
| ENSMUSG00000032554 | 1084.7700 | 305.8333 | 57.3967 | 9.5733 | -2.06 | 1.66E-08 | 1.40E-05 | down | Trf |
| ENSMUSG00000022474 | 426.0000 | 116.3333 | 28.8700 | 7.2333 | -2.08 | 2.08E-06 | 0.000877 | down | Pmm1 |
| ENSMUSG00000069805 | 3959.3333 | 1068.0000 | 219.8000 | 52.0733 | -2.10 | 2.07E-11 | 2.62E-08 | down | Fbp1 |
| ENSMUSG00000036216 | 1448.0000 | 386.0000 | 468.9667 | 126.2167 | -2.17 | 1.74E-05 | 0.004729 | down | Leap2 |
| XLOC_017750 | 314.0000 | 71.3333 | 7.5533 | 1.4967 | -2.29 | 2.37E-07 | 0.000135 | down | TCONS_00104968 |
| ENSMUSG00000030762 | 156.3333 | 36.6667 | 8.7733 | 1.7633 | -2.31 | 5.19E-05 | 0.012764 | down | Aqp8 |
| ENSMUSG00000066072 | 1395.7300 | 274.6667 | 49.0500 | 8.9100 | -2.51 | 1.53E-07 | 9.02E-05 | down | Cyp4a10 |
| ENSMUSG00000033898 | 15.9967 | 3.0033 | 0.7967 | 0.1467 | -2.54 | 0.000255 | 0.043068 | down | - |
| ENSMUSG00000070645 | 109.6667 | 21.6667 | 5.4233 | 0.9200 | -2.59 | 5.95E-05 | 0.014058 | down | Ren1 |
| XLOC_020942 | 98.6667 | 19.3333 | 7.6867 | 1.2833 | -2.62 | 0.000191 | 0.033877 | down | TCONS_00126047 |
| ENSMUSG00000042988 | 46.3333 | 8.0000 | 1.9867 | 0.3667 | -2.70 | 5.43E-06 | 0.001962 | down | Notum |
| ENSMUSG00000090486 | 10.0000 | 1.6667 | 0.2267 | 0.0333 | -2.71 | 0.000271 | 0.04439 | down | BC035947 |
| ENSMUSG00000021228 | 48.5067 | 8.3333 | 1.6667 | 0.2533 | -2.73 | 3.29E-08 | 2.43E-05 | down | Acot3 |
| ENSMUSG00000024266 | 30.3333 | 5.0000 | 1.2167 | 0.1767 | -2.76 | 6.32E-05 | 0.014741 | down | Adad2 |
| ENSMUSG00000037762 | 530.6667 | 83.6667 | 11.3267 | 1.6433 | -2.88 | 0.000137 | 0.027737 | down | Slc16a9 |
| XLOC_004875 | 35.0000 | 5.3333 | 31.6833 | 4.4567 | -2.90 | 7.22E-05 | 0.016394 | down | TCONS_00030167 |
| ENSMUSG00000059040 | 4143.2800 | 649.9900 | 178.5900 | 25.2400 | -2.91 | 2.75E-08 | 2.12E-05 | down | Eno1b |
| ENSMUSG00000072949 | 892.7933 | 134.6833 | 46.0400 | 6.1767 | -2.93 | 5.70E-21 | 5.05E-17 | down | Acot1 |
| ENSMUSG00000038641 | 19.6667 | 2.6667 | 1.3533 | 0.3800 | -3.06 | 4.59E-06 | 0.001693 | down | Akr1d1 |
| ENSMUSG00000029656 | 45.3333 | 5.6667 | 1.5233 | 0.1600 | -3.18 | 2.79E-05 | 0.007056 | down | C8b |
| ENSMUSG00000040808 | 362.0000 | 44.3333 | 185.6433 | 19.8433 | -3.27 | 8.31E-06 | 0.002678 | down | S100g |
| ENSMUSG00000037583 | 92.6667 | 10.6667 | 7.2800 | 0.7533 | -3.33 | 1.95E-11 | 2.62E-08 | down | Nr0b2 |
| ENSMUSG00000097239 | 24.9467 | 2.6633 | 0.9700 | 0.0900 | -3.45 | 0.000183 | 0.033326 | down | Gm27029 |
| ENSMUSG00000051225 | 76.3333 | 7.3333 | 4.8567 | 0.3100 | -3.55 | 0.000146 | 0.028806 | down | Fam83a |
| ENSMUSG00000055827 | 390.6900 | 35.2200 | 20.6900 | 1.1867 | -3.70 | 1.63E-05 | 0.004525 | down | Gsdmc3 |
| ENSMUSG00000056293 | 1869.0267 | 163.0233 | 65.6367 | 5.0133 | -3.78 | 3.52E-06 | 0.001355 | down | Gsdmc2 |
| ENSMUSG00000027875 | 5609.3333 | 471.6667 | 121.8100 | 8.8200 | -3.79 | 5.90E-18 | 3.48E-14 | down | Hmgcs2 |
| XLOC_011058 | 47.3333 | 3.6667 | 1.0933 | 0.0733 | -3.83 | 8.72E-08 | 5.46E-05 | down | TCONS_00057909 |
| ENSMUSG00000009588 | 394.0000 | 31.0000 | 12.0667 | 0.7867 | -3.92 | 1.65E-09 | 1.62E-06 | down | St6galnac1 |
| ENSMUSG00000090268 | 105.6667 | 6.6667 | 5.3000 | 0.2967 | -4.16 | 8.34E-13 | 1.34E-09 | down | Gm5286 |
| ENSMUSG00000064225 | 1086.6667 | 64.6667 | 8.5333 | 0.4333 | -4.27 | 3.04E-13 | 5.38E-10 | down | Paqr9 |
| ENSMUSG00000009633 | 662.6667 | 39.0000 | 76.4900 | 4.2433 | -4.33 | 1.00E-13 | 1.98E-10 | down | G0s2 |
| ENSMUSG00000053388 | 20.0000 | 1.0000 | 0.9633 | 0.0533 | -4.34 | 1.89E-06 | 0.000836 | down | Trim50 |
| ENSMUSG00000059201 | 24.6667 | 1.0000 | 0.6867 | 0.0200 | -4.63 | 2.04E-09 | 1.90E-06 | down | Lep |
| ENSMUSG00000005681 | 230.0000 | 6.6667 | 91.3700 | 2.6767 | -5.30 | 4.38E-08 | 2.99E-05 | down | Apoa2 |
| ENSMUSG00000022650 | 76.6667 | 1.3333 | 14.9267 | 0.2033 | -5.94 | 6.62E-05 | 0.015223 | down | Retnlb |
| ENSMUSG00000079025 | 27.4400 | 0.3333 | 0.7900 | 0.0100 | -6.16 | 9.02E-05 | 0.019489 | down | Gsdmc |
| XLOC_002901 | 9.3333 | 0.0000 | 6.7500 | 0.0010 | -6.40 | 0.000173 | 0.032555 | down | TCONS_00019039 |
| ENSMUSG00000078746 | 10.5467 | 0.0000 | 0.1700 | 0.0010 | -6.56 | 0.000179 | 0.032991 | down | Fam205a4 |
| XLOC_005084 | 14.6000 | 0.0000 | 1.4200 | 0.0010 | -6.97 | 0.000122 | 0.026074 | down | TCONS_00030454 |
| ENSMUSG00000093485 | 36.5533 | 0.0000 | 1.5233 | 0.0010 | -8.35 | 3.01E-06 | 0.001184 | down | Gm20708 |
| ENSMUSG00000109941 | 76.5900 | 0.0000 | 4.5167 | 0.0010 | -9.46 | 0.000143 | 0.028562 | down | Exosc6 |
| ENSMUSG00000030653 | 108.6267 | 0.0000 | 1.8267 | 0.0010 | -9.84 | 4.72E-08 | 3.10E-05 | down | Pde2a |
| ENSMUSG00000066878 | 836.9567 | 0.0000 | 31.8733 | 0.0010 | -12.87 | 3.35E-12 | 4.95E-09 | down | Gm10184 |
