# Supplementary figures and images for: A diet high in sugar and fat influences neurotransmitter metabolism and then affects brain function by altering the gut microbiota
Source: Transl Psychiatry. 2021 May 27;11:328. doi: 10.1038/s41398-021-01443-2 (PMC8160265; doi:10.1038/s41398-021-01443-2)

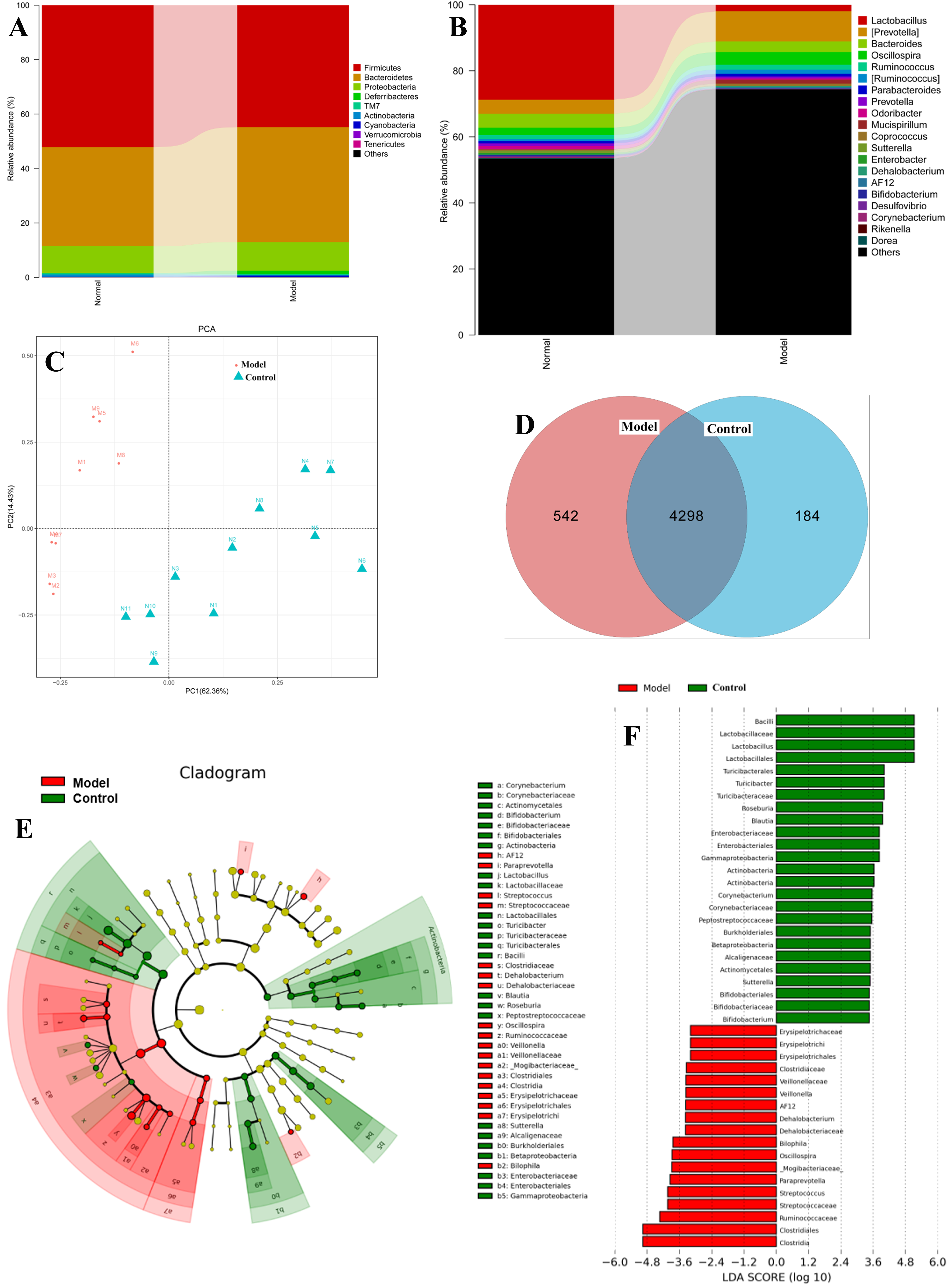

Supplement: Supplementary file 2 — Figure S1 [file 41398_2021_1443_MOESM2_ESM.tif]

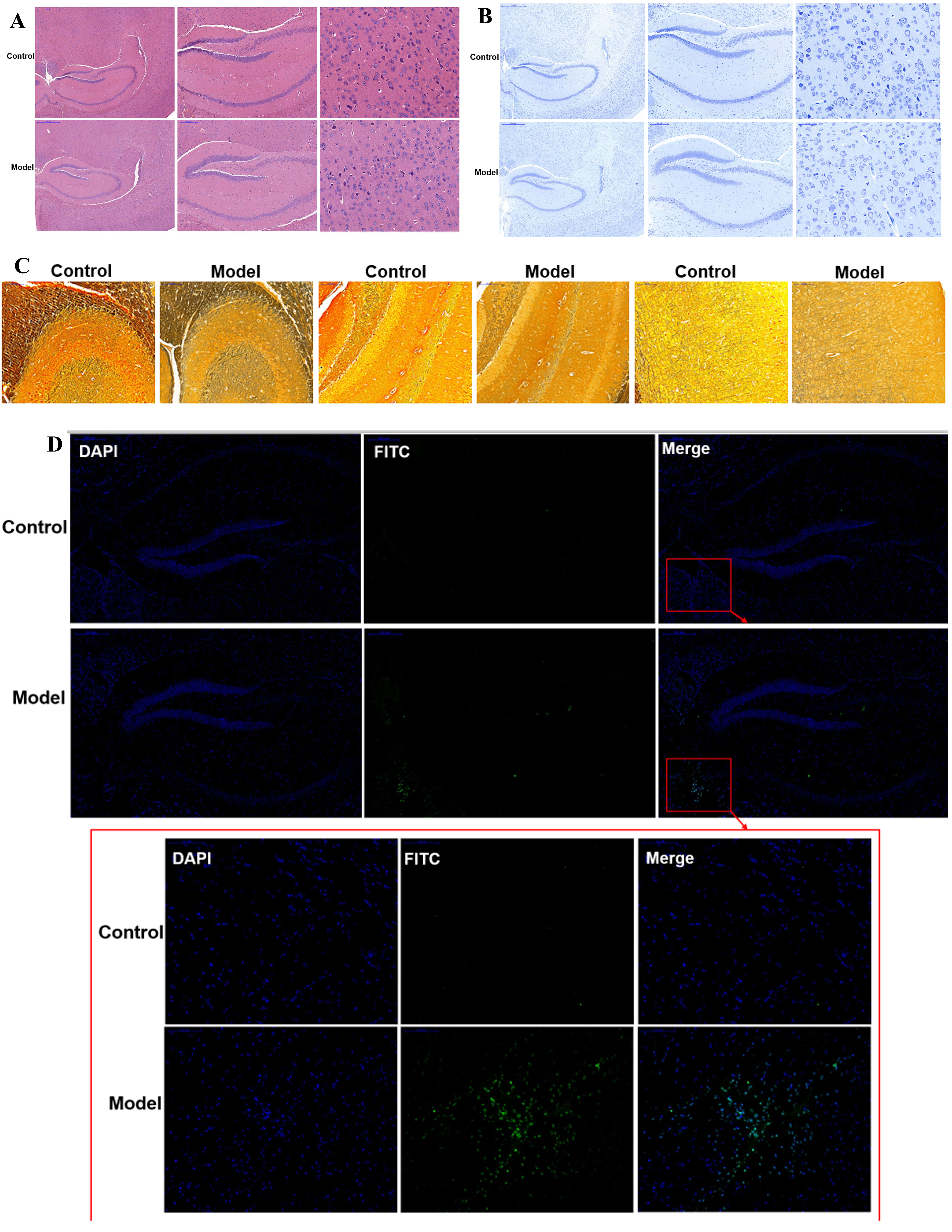

Supplement: Supplementary file 4 — Figure S3A [file 41398_2021_1443_MOESM4_ESM.tif]

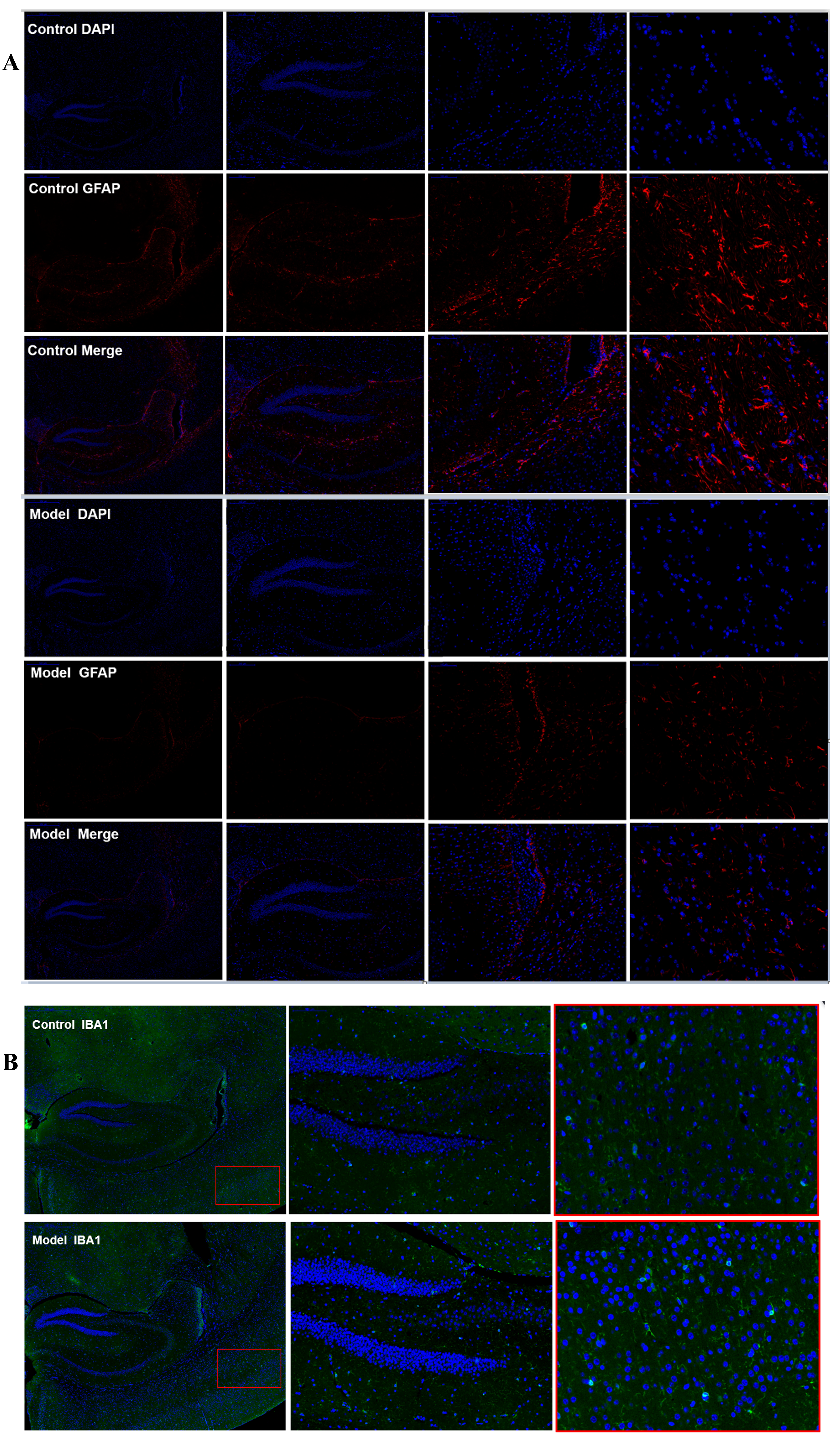

Supplement: Supplementary file 5 — Figure S3B [file 41398_2021_1443_MOESM5_ESM.tif]

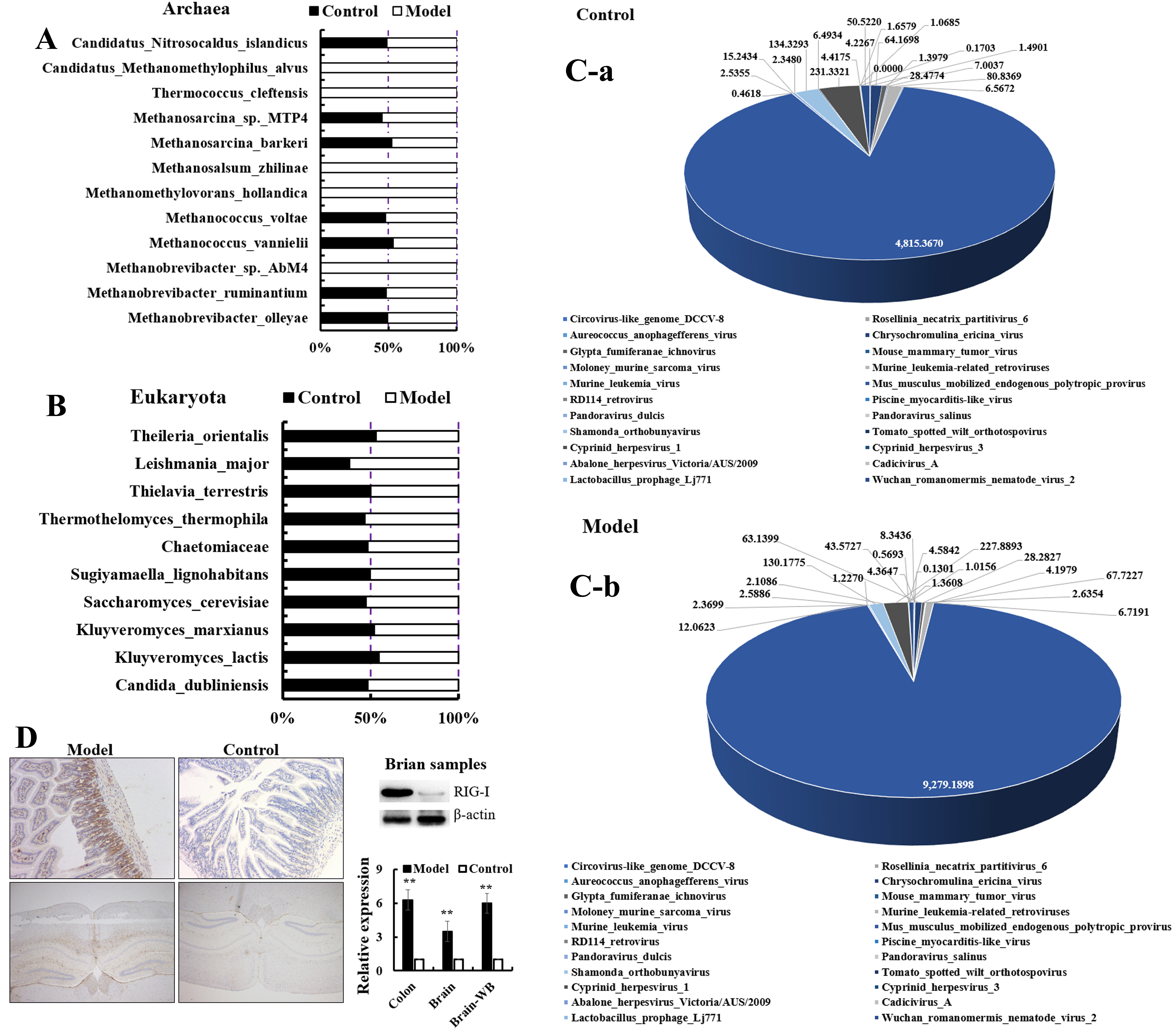

Supplement: Supplementary file 6 — Figure S4 [file 41398_2021_1443_MOESM6_ESM.tif]

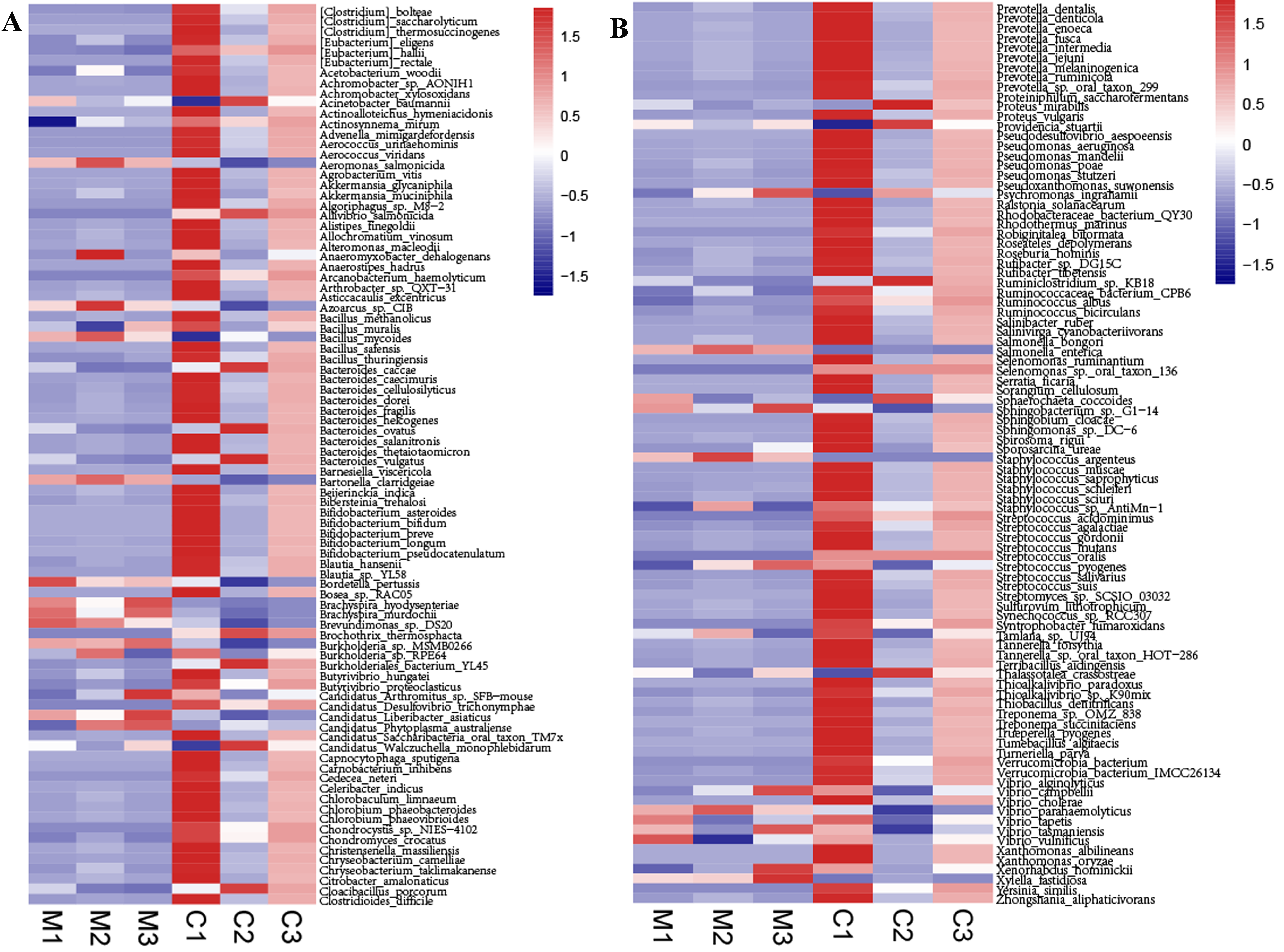

Supplement: Supplementary file 7 — Figure S5A-B [file 41398_2021_1443_MOESM7_ESM.tif]

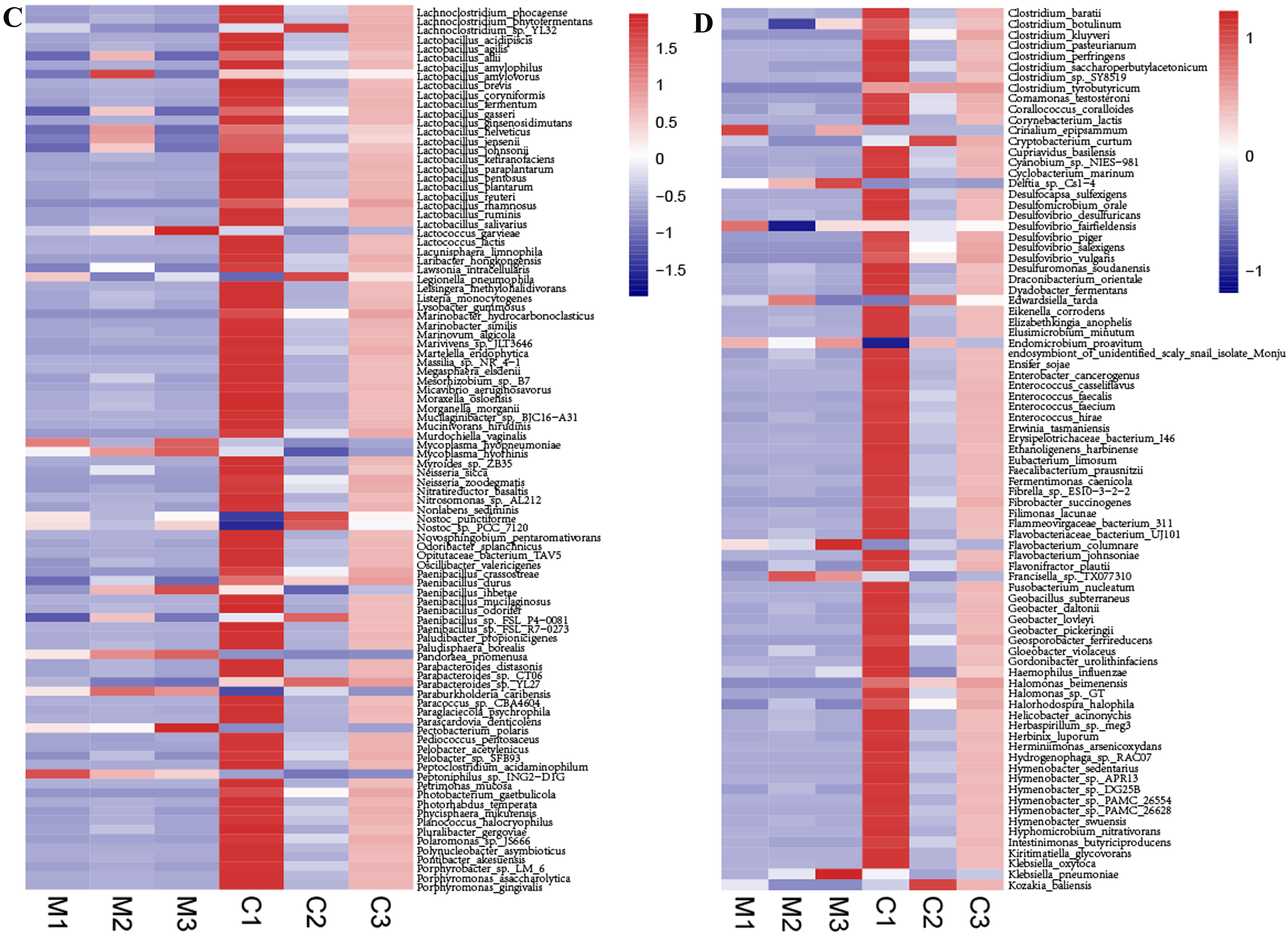

Supplement: Supplementary file 8 — Figure S5C-D [file 41398_2021_1443_MOESM8_ESM.tif]
